# Protocol of the digital long COVID study: A single-center, registry-based, feasibility and clinical evaluation study to investigate a 12-week digital intervention program for people affected by post-COVID-19 condition

**DOI:** 10.1371/journal.pone.0340385

**Published:** 2026-01-20

**Authors:** Stefan Rohner, Rebekka Schnepper, Andrea Meienberg, Katrin Bopp, Michael Mayr, Gunther Meinlschmidt, Rainer Schaefert

**Affiliations:** 1 Faculty of Medicine, University of Basel, Basel, Switzerland; 2 Department of Psychosomatic Medicine, University Hospital Basel, Basel, Switzerland; 3 Department of Digital and Blended Psychosomatics and Psychotherapy, University Hospital Basel, Basel, Switzerland; 4 Medical Outpatient Clinic, University Hospital Basel, Basel, Switzerland; 5 Department of Clinical Psychology and Psychotherapy – Methods and Approaches, Trier University, Trier, Germany; Wingate University, UNITED STATES OF AMERICA

## Abstract

Up to 400 million individuals globally are estimated to experience persistent symptoms, including fatigue, muscle pain, and brain fog, following severe acute respiratory syndrome coronavirus type 2 infection. These persistent symptoms are referred to as Post-COVID-19 condition if they last for more than 12 weeks after infection and persist for at least 8 weeks and often causing significant distress and burden. The underlying pathological mechanisms have not yet been fully elucidated. Due to the heterogeneity of the disease a multifactorial origin is highly likely. Overall, evidence on optimal management is limited, and no medication has yet proven to be effective. Current symptom management and treatment guidelines suggest a biopsychosocial perspective and emphasize multidisciplinary approaches. Comprehensive interventions, adequate treatment access, and appropriate resources remain insufficiently available and implementing digital interventions might help mitigate these limitations. This protocol details a single-site feasibility and clinical evaluation study aiming to bridge this gap. By implementing an exploratory, open-label, digital interventional approach this study investigates the feasibility and efficacy of a 12-week program delivered by a cloud-based application. The program consists of 13 modules encompassing a wide range of topics (e.g., energy management, self-care, stress management) and includes informational (e.g., psychoeducational content) and interactive (e.g., exercises, self-reflection diaries) components. Customization options align the material with participant needs. A dedicated feedback section in each module captures feedback regarding usability and feasibility. Participants are monitored and checked for adherence throughout the study. The primary outcome is the post-intervention change in functional capacity measured by the World Health Organization Disability Assessment Schedule 2.0. All participants provide written informed consent. Key results from the study will be published in peer-reviewed journals.

## Introduction

### Background

An estimated 400 million individuals experience persistent symptoms following a severe acute respiratory syndrome coronavirus type 2 (SARS-CoV-2) infection [1], significantly impacting their daily lives. The prevalence of self-reported symptoms with functional limitations attributed to the coronavirus disease 2019 (COVID-19) is estimated at 1.2%–4.8% after 12 weeks [2]. The sum of these persistent symptoms has been referred to as Long COVID, post-acute sequelae of SARS-CoV-2 infection, post-COVID syndrome, or Post-COVID-19 condition (PCC) without a clear consensus in the literature [3,4]. Acknowledging that these terms are often used interchangeably, we will primarily use PCC to refer to the persistent symptoms individuals experience after a SARS-CoV-2 infection. Various symptoms have been reported to be associated with PCC and include fatigue, cough, shortness of breath, difficulty concentrating, altered taste, sleep disorders, myalgia, chest pain, and palpitations [5–7]. This spectrum of symptoms indicates the involvement of multiple organ systems [8]. Various theories regarding the pathogenesis and predictive factors of PCC have received attention. Recent reviews report a) altered immune response, b) microbiota dysregulation, c) autoimmunity as potential pathophysiological explanations [9], and d) depression and anxiety as PCC predictors [10]. Furthermore, results of a French cross-sectional study showed a higher positive association between persistent physical symptoms and the belief of a having experienced COVID-19 than with a laboratory confirmed infection [11]. Another study has shown, that persistent symptoms are reported slightly more in COVID-19 confirmed cases than without such confirmation [12]. The challenge of PCC can be summarized as the lack of robust explanations regarding the manifestation and fluctuation of symptoms, since the presentation of symptoms is influenced not only by biological, but by psychological and social factors as well [13]. This challenge is being addressed through comprehensive treatment approaches [14,15] and current management and care recommendations that integrate various medical specialties [16–18], supported by emerging evidence on the effectiveness of multifaceted interventions [e.g., 19–21].

A systematic review evaluated the effectiveness of treatments and interventions for PCC [22]. It concludes that there is no strong evidence supporting drug therapies, hyperbaric oxygen therapy, and various dietary supplements. However, moderate evidence suggests that online cognitive behavioral therapy may help to reduce fatigue, a supervised online rehabilitation program combining physical and mental health could improve overall quality of life, and intermittent aerobic exercise may enhance physical function.

### Research gap

Comprehensive, interdisciplinary treatment options for PCC remain scarce in Switzerland, underscoring the urgent need for multimodal approaches. Digital interventions offer a promising way to address resource constraints and improve accessibility by providing remote, tailored treatments, including remote monitoring, symptom tracking, and individualized symptom management [23–26]. However, evidence on their effectiveness is mixed: a randomized trial using a videogame interface showed no improvement in cognitive symptoms [27], while a systematic review and meta-analysis of telerehabilitation highlighted gains in physical function but little impact on clinical or psychosocial outcomes [28]. Conversely, a scoping review found overall benefits for various digital interventions, especially those combining physical and psychological components [29], with one study demonstrating the effectiveness of integrating sleep, stress management, and energy conservation into a broader program [30]. Despite these promising findings, concerns about the lack of theory-based research, inconsistent results, and low adherence rates persist [29,30], emphasizing the need for a multidisciplinary strategy that unites physical and psychological interventions to optimize care and outcomes.

### Rationale

To address the current lack of comprehensive digital interventions, the Digital Long COVID Study (DiLCoS) integrates physical (e.g., evidence-based relaxation techniques such as progressive muscle relaxation, dietary guidance) and psychological components (e.g., cognitive distancing, mindfulness, and acceptance-based strategies). Recognizing that PCC presents a complex spectrum of symptoms requiring a multidisciplinary approach DiLCoS applies a biopsychosocial perspective to symptom management, combining various evidence-based interventions to improve patient outcomes. Dedicated feedback sections systematically gather user insights on feasibility and usability, ensuring continuous optimization, while regular adherence checks and data monitoring enhance engagement and promote high completion rates. This rationale is supported by the following key considerations.

- Digital format and accessibility: The digital format ensures greater accessibility and convenience for participants. By providing resources through a cloud-based application, DiLCoS ensures that support and guidance are readily available, overcoming geographical and physical barriers that often limit access to care. Notably, the application includes a text-based chat function, enabling effective and efficient communication with the study team.
- Dual approach: The integration of both informational (e.g., psychoeducational content) and interactive (e.g., exercises, self-reflection diaries) components in the application ensures a dynamic and engaging intervention. This dual approach is crucial in managing chronic symptoms, as it aids adapting to the participant’s condition and promotes self-management [31].
- Evidence-based content and structure: The content and structure of DiLCoS are grounded in the latest research and insights from successful past digital interventions for PCC. Each module targets specific domains of PCC, drawing on evidence-based practices for symptom management and improvement [e.g., 19–21].

### Objectives and trial design

DiLCoS aims to evaluate the feasibility and efficacy of a digital intervention for PCC and to assess the practical implementation of the 12-week program as well as its initial evidence in a clinical setting. Designed as a feasibility and clinical evaluation study it measures outcomes before and after the intervention with functional disability of main interest (primary outcome), feasibility and usability parameters of the material and delivery platform (e.g., user satisfaction, perceived helpfulness, ease of use), and symptom severity and persistence, well-being and quality of life, as well as markers of depression and anxiety (additional secondary outcomes). Following an open-label, registry-based design approach, all interested and eligible individuals are recruited. Participants from the Basel Long COVID Cohort Study (BALCoS) are planned to be considered a non-randomized control group, as they will have completed the same measurements and assessments as the DiLCoS participants, without the intervention and its evaluation (see statistical methods for further details). No formal matching or randomization will be performed. However, we will compare baseline (BL) characteristics (e.g., age, sex, PCC symptom severity, duration of PCC symptoms at BL between DiLCoS and BALCoS participants to identify and adjust for potential confounding variables in the analysis.

### Horizon Europe long COVID project

The DiLCoS study is part of the Horizon Europe Long COVID project [32] coordinated by Helsinki University Hospital. This project brings together leading expertise from clinical medicine, virology, metabolism, and immunology to investigate the mechanisms of PCC. Adopting a biopsychosocial perspective, the project aims to develop a comprehensive understanding of PCC by identifying underlying mechanisms, processes, and biomarkers, as well as effective management and treatment strategies. Research efforts include cohort studies, biomechanistic studies, and digital intervention studies, with DiLCoS being one of the latter.

## Methods

### Ethics statement

The study has been approved by the Department of Clinical Research at University Hospital Basel (ID: th22schaefert), the Ethics Commission of Northwest and Central Switzerland (ID: 2023−00359) and is registered at ClinicalTrial.gov (ID: NCT05781893). All participants provide written informed consent.

### Sample size

A power analysis was performed using G*Power 3.1 [33] to determine the minimum number of required study participants to detect an effect size of Cohen’s f = 0.15 in the primary outcome of functional capacity using the World Health Organization Disability Schedule (WHODAS 2.0) – measured before and after the intervention. The calculation was based on: 1) repeated measures, within factors ANOVA, 2) two timepoints (pre-post comparison), 3) power (1- ß) of 0.80, 4) α = 0.05, and 5) a small to medium effect size of f = 0.15, derived from recent reviews [22,29,34] on digital and non-digital interventions for PCC.

The result indicated a target sample size of N = 105, including a 15% allowance for dropouts. Interim enrolment progress is monitored to ensure this target is met.

### Study setting and recruitment

This single-site study is conducted at the University Hospital Basel (UHB), where all study-related activities – including screening, informed consent, onboarding, and data collection – take place. While some participants are recruited internally from patients attending the PCC consultation at the Medical Outpatient Clinic of the UHB, others are recruited externally, with procedures for the latter group conducted remotely when necessary. All inquiries from interested individuals, whether internal or external, are directed to and managed by the study team at the UHB independently. DiLCoS collects data mainly remotely from participants living in Switzerland, Germany, and Austria. Recruitment procedures for DiLCoS are identical to those for BALCoS. These recruitment procedures are outlined in the published study protocol [35], except the expanded recruitment strategies outlined in our respective ethics amendments. Expanded advertising and recruitment strategies include:

- PCC consultation services outside UHB, clinics or participant pools of PCC studies from the German-speaking part of Switzerland, Germany, and Austria, are contacted to distribute study advertisements. This includes displaying posters and flyers in waiting rooms and handing out flyers to patients after consultations. Their involvement is strictly limited to advertising purposes – they do not participate in or contribute to any research activities.
- Posting study advertisements in patient networks, releasing press statements, and putting up posters in public buildings and spaces, and public transport.
- Advertising the study online (i.e., on Instagram and via Google Ads).

All potential participants are screened by the study team and included based on the following eligibility criteria (Table 1). Recruitment for the study has started 1^st^ February 2024 and is expected to conclude 31^st^ March 2025. Data collection is expected to be finished 30^th^ June 2025 and we expect results of the study to be reportable in March 2026.

**Table 1:** Eligibility criteria.

Inclusion criteria	Exclusion criteria
Confirmed Post-COVID-19 condition diagnosis or subjective attribution of symptoms to Post-COVID-19 condition	Lack of general understanding of and/ or inability to adhere to study procedures
Participation in the Basel Long COVID Cohort Study	Rejection of consent to participate
Possession of smartphone or tablet device capable of running the application	

According to World Health Organization guidelines PCC will be defined as [36]:

- History of probable or confirmed SARS-CoV-2 infection.
- Symptoms cannot be explained by an alternative diagnosis.
- Symptoms usually start within 3 months after the onset of the acute SARS-CoV-2 infection and with symptoms and effects last usually for at least 2 months.

### Participants timeline

**Fig 1:**
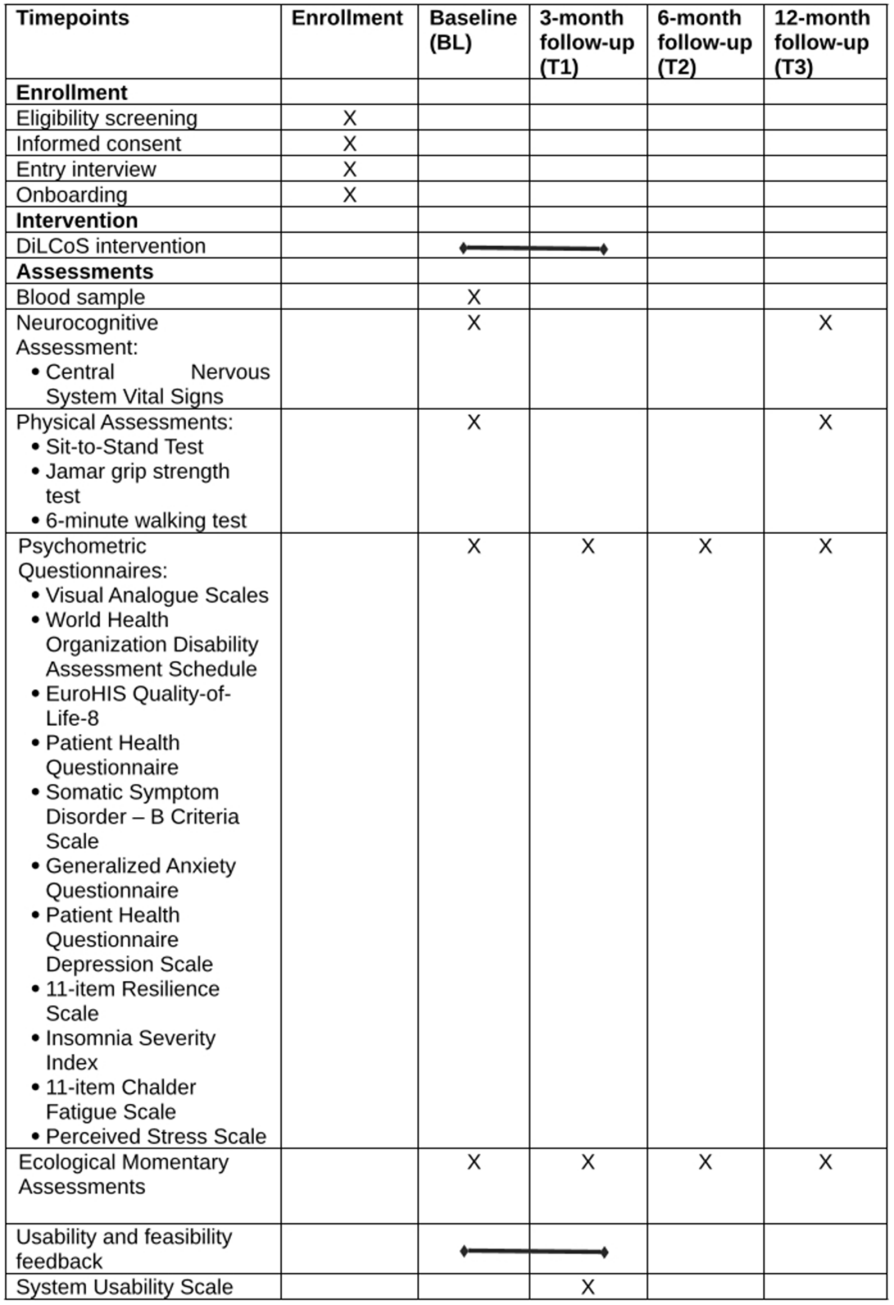
Study schedule. Schedule of enrollment, interventions, and assessments of the Basel Long COVID Cohort Study (BALCoS) and the Digital Long COVID Study (DiLCoS).

The study team screens potential participants based on eligibility criteria (Table 1) and obtains written informed consent, which is required for participation. Informed consent for BALCoS and DiLCoS are routinely obtained together. The informed consent forms are completed by hand or mailed in. The DiLCoS form includes two provisions: 1) consent to participate in the study, and 2) consent to allow anonymized data collected within DiLCoS to be used in future research projects. The study team explains to each participant the purpose of the study, procedures, the expected duration, potential risks and benefits, and possible inconveniences. Specifically, that participants might reduce the frequency of existing accompanying therapies to accommodate study participation. However, no therapy or medication is prohibited during the intervention. Participants are explicitly informed that participation is voluntary and withdrawal from the study is possible at any time without a reason. The study team and participant coordinate the entry interview that covers clinical information (e.g., symptoms, vaccination schedules, therapies, and treatments; see the available pre-print of the BALCoS study protocol for more details). After the interview participants complete the onboarding process. Onboarding is conducted either in person or via telephone/video calls. Participants are added to the cloud-based platform, and the study team explains its functionality and navigation features. A demonstration module introduces the basic module structure (see intervention for details). Study processes and interactions, such as notifications, daily material, and the overall study timeline, are explained. Participants are informed that all exercises are voluntary and can be omitted, interrupted, or resumed later. The intervention begins after the BALCoS BL measurements are completed and usually within two weeks after onboarding. Within DiLCoS, participants complete BL assessments, followed by post-intervention assessments T1 (3 months post-BL). Assessments at T2 (6 months post-BL) and T3 (12 months post-BL) are part of the overarching BALCoS schedule. However, because T2 is not an active follow-up point for DiLCoS-specific outcomes, DiLCoS participants will only be compared with BALCoS participants at T1 and T3 for secondary and additional outcomes (Fig 1 and S4 Fig).

### The DiLCoS intervention

#### Development of the DiLCoS intervention.

The development of the intervention has been informed by the newest available literature and in collaboration with expert groups inside the UHB (e.g., physiotherapists, dieticians, physicians, psychotherapists, and psychologists), An early version of the implemented material and the application was reviewed with patients consulting the specialized PCC clinic at UHB. Comments and feedback were reviewed and discussed by the study team and taken into consideration during the development of the application and the material. Additionally, the information of patients visiting the specialized PCC clinic was taken into account regarding what specific areas they lack knowledge or would wish for specific instruments or guidance.

#### Rationale and modules.

Following the biopsychosocial understanding of PCC, the intervention encompasses a broad spectrum of topics. These topics are organized in 13 modules (Table 2).

**Table 2:** Rationale and summary of the 13 modules.

Module number	Title	Summary/ Rationale
0	Introduction	Orientation to the intervention platform, brief overview of current knowledge about Post-COVID-19 condition (PCC), and the biopsychosocial model of PCC as the rationale for a multimodal approach
1	Energy Management	Introduction to basic energy management, fatigue, Post-Exertional Malaises, and flare-ups
2	Healthy Lifestyle	Basic dietary guidelines aligned with existing post-viral recovery recommendation (Mediterranean diet), recommendations for reflection and change of media consumption behavior
3	Physical Activity	Focus on fatigue and fatigue mitigation techniques informed by chronic illness rehabilitation research, basic pacing guidance, introduction to different breathing techniques and their execution
4	Wrap-Up Week 1	Recap of the most useful and effective exercises from Modules 1–3 based on user feedback
5	Mental Health	Introduction to PCC-related mental health problems, focus on mental and physical self-management, incorporating social and community support strategies, exercises drawn from cognitive-behavioral therapy
6	Acceptance	Focus on acceptance and commitment therapy elements to facilitate reflection on illness-related life situation and foster a positive outlook, aligned with current therapeutic approaches to chronic illness
7	Self-Care	Introduction to self-care and self-compassion, guidance on practical self-compassion exercises, recommendations for self-care activities to foster positive experience and coping strategies
8	Wrap-Up Week 2	Recap of the most useful and effective exercises from Modules 5–7 based on user feedback
9 A	Sleep	Tailored content based on individual preference: focus on sleep disturbances and sleep hygiene, guidance on techniques drawn from cognitive-behavioral therapy to promote healthy sleeping behavior
9 B	Neurocognitive Impairment	Tailored content based on individual preference: focus on strategies, techniques, and exercises to improve cognitive function (memory, concentration, executive function)
9 C	Pain Management	Tailored content based on individual preference: focus on strategies and techniques for pain relief and improvement of quality of life, recap of relaxation techniques and breathing exercises from previous modules
10	Stress Management	Relaxation techniques (e.g., progressive muscle relaxation), introduction to positive and negative stress (eustress – distress), techniques drawn from cognitive-behavioral therapy to promote positive thinking regarding stress
11	Motivation and Hope	Introduction to predictive coding, focus on subjective expectations regarding PCC symptoms, and recommendations for coping strategies to deal with fluctuating symptoms
12	Conclusion	Final summary, reflection on intervention, next-step recommendations for implementing

#### Structure of the DiLCoS intervention.

**Fig 2:**
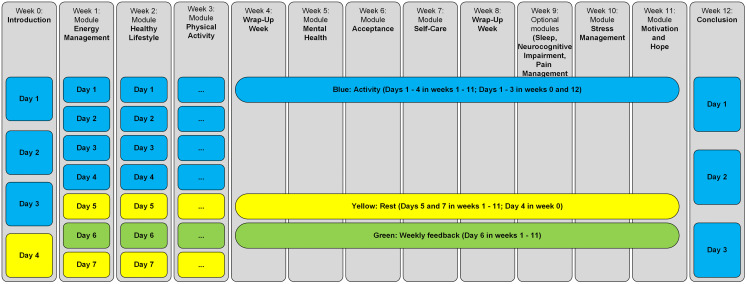
Structure of the DiLCoS intervention.

Modules include an introduction, a summary, and interactive (i.e., exercises) and information (i.e., psychoeducational) elements, as well as a dedicated feedback section. Most modules last 7 days except for module 0: Introduction (4 days) and module 12: Conclusion (3 days). The expected daily time commitment is approximately 15 minutes depending on module volume. All modules (except the conclusion module 12) include at least one rest day. Days marked in blue (activity days) include interactive content containing multi-media files, portable document formats, exercises, and written content. The dedicated feedback section is marked in green (Day 6 of the module). Planned rest days are marked in yellow (Fig 2).

#### Individualization.

In order to incorporate individual preferences regarding the content of the intervention, the intervention deploys two mechanisms:

- Two of the modules (week 4 and 8) are so called “Wrap-Up Weeks”, where participants receive minimal new informational elements and the interactive elements consist of the exercises that participants scored highest in terms of helpfulness and liking in the preceding three modules (modules 1–3 for week 4 and modules 5–7 for week 8, evaluations collected on day 6 respectively).
- Participants chose one out of three topics for module 9 at the time of onboarding: A – sleep, B – neurocognitive impairment, C – pain management, with option A as default. The study team determined that the topic of sleep might benefit all participants, regardless of presence or absence of sleep disturbances.

#### Technical solution.

The program is delivered by the Docdok.health system [37] – a cloud-based interactive platform that includes a smartphone and tablet application over which participants receive the entire intervention automatically. The system allows participants to engage in the daily material at their own pace, with only an internet connection and a mobile device (smartphone or tablet) required to access the intervention content. Participants receive a notification from the application each time a new module is available (once a week) and a daily reminder at 5 p.m. if the daily content has not been completed. The mobile application includes a text chat to facilitate communication between participants and study personnel.

### Data collection and security

Data will be collected through two primary sources: 1) Participants will enter data into the Docdok.health system via the application, and 2) additional study data will be recorded in the Electronic Data Capture System [38]. The latter is a cloud-based database hosted by UHB, where BL and follow-up data are recorded. Participants can access the questionnaires via a link and a login code sent either manually by the study team or automatically by the system. Some BL data (e.g., demographic information, neurocognitive assessment) and some T3 data (e.g., neurocognitive assessment, physical performance scores) are entered by the study team. For T1 and T2 data, participants receive access information in an automatically generated email from the system after the intervention is completed. If participants have not completed these follow-up measurements after two weeks, they are contacted by the study team. Please see the published study protocol for the BALCoS study for further details on the follow-up procedures [38].

All collected data will be encoded before any further analysis. Logins for both the Docdok.health system and the UHB-hosted database are individualized and require a password or code. These measures ensure data safety and traceability of access for both data collection tools. Data from the Docdok.health system and the UHB-hosted database are stored on servers located in Switzerland, where Docdok.health is registered, and on UHB servers in Basel, Switzerland respectively.

### Outcomes

#### Primary outcome.

The primary outcome for DiLCoS is the change in participants’ functional capacity measured by the World Health Organization Disability Assessment 2.0 [WHODAS 2.0, 12-item version; 39] from BL to T1. This measure was selected as the primary outcome measure due to its widespread validation in assessing functional capacity across multiple domains and its suitability for complex, multisystemic conditions like PCC [40–42]. It is recommended for capturing disability and functional impairment in both post-acute conditions and chronic illnesses, making it highly relevant for measuring the broad impact of PCC on daily life.

#### Feasibility and usability outcomes.

The feasibility and usability of the intervention and its delivery platform will be evaluated through both quantitative and qualitative feedback collected within the intervention. Additionally, usability will be assessed using the System Usability Scale [SUS; 43], administered at T1. The intervention will be considered feasible if the following thresholds are met: 1) completion rate of at least 50%, and 2) dropout rate remains below 15%. Usability will be supported if the average system usability score exceeds the acceptable threshold of 68 [44]. Of further consideration are these key parameters: a) user satisfaction b) perceived duration, c) content volume, d) perceived helpfulness, e) ease of use, and f) challenges or barriers regarding the material. These aspects will be measured through both quantitative ratings and qualitative feedback to ensure a comprehensive understanding of user experience.

- Quantitative feedback is gathered through Likert-scaled questions (e.g., “How did you like the exercises in this module?” rated from 1 = “not at all” to 5 = “very much”).
- Qualitative feedback is collected via open-ended text responses, allowing participants to elaborate on their experiences. A structured content analysis [45] will be conducted to identify common themes and areas for improvement.

This evaluation will determine whether the DiLCoS intervention is acceptable and implementable on a broader scale.

#### Further secondary outcomes.

Further secondary outcomes will assess changes in additional psychometric questionnaires administered within BALCoS from BL to T1. These measures are (see Fig 1 for an overview):

- Visual Analogue Scales [VAS; 46] regarding symptom intensity, functional impairment, quality of life, and work capacity.
- Quality of Life: EuroHIS Quality-of- Life-8 [QOL-8; 47]
- Somatic Symptom Severity: Patient Health Questionnaire [PHQ-15; 48]
- Somatic Distress: Somatic Symptom Disorder – B criteria scale [SSD-12; 49]
- Depression: Patient Health Questionnaire Depression Scale [PHQ-8; 50]
- Anxiety: Generalized Anxiety Disorder Questionnaire [GAD-7; 51]
- Resilience: 11-item Resilience Scale [RS-11; 52]
- Insomnia: Insomnia Severity Index [ISI; 53]
- Fatigue: 11-item Chalder Fatigue Scale [CFS; 54]
- Stress: Perceived Stress Scale [PSS-10; 55]
- Ecological Momentary Assessment (EMA): 8 items total, Items 1–5 are based on the Screening Tool for Psychological Distress [STOP-D; 56], Item 6 is based on the European Quality of Life 5 Dimensions [EQ-5D; 57, 58], Item 7 is based on the Single Item Mindfulness Scale [SIMS; 59], and Item 8 is based on the Single Item Self-Compassion Scale [SISC; 60].

Further secondary outcomes include changes in physical performance and neurocognitive assessment scores from BL to T3 as measured within BALCoS by the sit-to-stand test [STS-60; 61], the Jamar grip strength test [62], the 6-minute walking test [63], and Central Nervous System Vital Signs [CNSVS; 64].

### Statistical methods

#### Descriptive statistics.

We will describe participant demographics, BL characteristics, and feasibility outcomes (e.g., completion and dropout rate) using appropriate measures of central tendency (mean or median) and dispersion (standard deviation or interquartile range), depending on variable distribution for continuous variables, and proportions (with 95% confidence interval) for categorical outcomes.

#### Inferential statistics.

We will conduct intention-to-treat analyses including all enrolled participants, complemented by per-protocol (completer) analyses that will include only participants who completed at least 50% of the total intervention modules. We will conduct pre-post comparisons of the primary and secondary outcomes, with BL as the pre-intervention measurement for which to adjust, and T1 as the post-intervention measurement. We will apply linear mixed-effects models to estimate changes in continuous outcomes (e.g., fatigue, quality of life), adjusting for potential confounders such as gender, age, and comorbidities. We will apply logistic regression or variations thereof to analyze dichotomous feasibility outcomes. Generalized estimating equations may be applied for repeated categorical outcomes.

#### Comparative analysis.

We will compare DiLCoS with BALCoS, using BALCoS-only participants as a nonrandomized control group. Eligibility, index dates, and follow-up windows will be aligned across cohorts. Propensity scores will be estimated from pre-index covariates such as demographics, BL clinical severity, site, and calendar time (e.g., enrollment quarter) to account for period effects. Stabilized inverse-probability-of-treatment weights (IPTW) shall be applied; common support will be enforced, and extreme weights may be truncated. Covariate balance will be assessed using absolute standardized mean differences (SMD, target <0.10). Outcomes shall be estimated with weighted generalized linear or Cox models with robust (sandwich) standard errors; for repeated measures, weighted Generalized Estimating Equations (GEE) or mixed-effects models will be used as appropriate. Sensitivity analyses will include propensity-score matching (such as 1:k nearest neighbor within a 0.2 Standard Deviation (SD) caliper of the logit propensity score) and assessments of robustness to unmeasured confounding (such as Rosenbaum bounds or E-values).

#### Non-parametric and effect size analyses.

When parametric assumptions are not met, we will use rank-based procedures matched to the design. We will use Wilcoxon signed-rank text for paired comparisons, Mann-Whitney U tests for two independent groups, and for repeated/factorial designs we will use Brunner-Langer ANOVA-type statistics (ATS) or rank-based linear mixed models (LMMs) for treatment x time effects. Effect sizes will include Hedges’ g (small-sample bias correction) and where appropriate odds ratios for binary outcomes, and relative-treatment effect (RTE) for rank-based analyses. All analyses will be employed with a 95% confidence interval.

#### Additional exploratory analyses.

Potential interactions (e.g., group × time, sex × symptom severity) may be modeled to examine differences in outcome trajectories between intervention and control participants.

#### Handling of missing data.

Missing item-level data will be addressed using multiple imputation by chained equations (MICE) under a missing-at-random assumption in the primary analysis.

Diagnostics shall include respective procedures, such as fraction of missing information (FMI), Monte-Carlo error, and distribution overlays of observed and imputed values. Sensitivity analyses will assess departures from missing at random (MAR), e.g., using δ-adjusted pattern-mixture models).

#### Benchmarking-controlled trial approach.

The results of our analyses may also serve as an additional comparator arm in another intervention trial conducted within the Horizon Europe Long COVID project (registered on ClinicalTrials.gov, ID: NCT05212467) and vice versa within a benchmark-controlled trial design [65].

For benchmarking comparisons, we will use propensity-score matching to mitigate BL confounding. We will harmonize eligibility, index dates, and follow-up across sources; estimate propensity scores from pre-index covariates using models such as logistic regression or machine learning; restrict to common support; and match treated to benchmark controls (e.g., nearest neighbor within a caliper/ratio), excluding unmatched units. Covariate balance will be assessed using metrics such as standardized mean differences before estimating outcomes with matched-data methods such as stratified Cox or conditional logistic models; sensitivity analyses will vary matching specifications (e.g., caliper, ratio) and apply procedures such as trimming.

#### Mixed methods.

We will use a mixed-methods approach to evaluate feasibility and usability outcomes by combining quantitative statistical procedures as outlined above (descriptive and inferential) and qualitative analysis in the form of a structured content analysis [45]. The content analysis draws on the richness of qualitative data captured in the Docdok.health application in textboxes throughout the intervention and the written communication channels (e.g., text chat or email). Where appropriate, we will include feedback, provided in telephone conversations, as documented by the study team. The coding framework consists of 1) *a priori* determined key usability and feasibility aspects (user satisfaction, perceived duration, content volume, perceived helpfulness, ease of use, and challenges or barriers) and 2) open coding, which allows capturing emerging themes in the data. Coding strategies adopted from Saldana [66] are outlined below (Table 3).

**Table 3:** Coding strategies for qualitative analysis.

Coding Framework	First Cycle Coding	Second Cycle Coding	Remarks
A priori determined usability and feasibility aspects	Descriptive coding	Pattern coding	A priori aspects function as categories for coding
Open coding	Descriptive coding	Pattern coding	Categories are synthesized from emerging themes

As our study recruitment is planned to be completed before qualitative data analysis is conducted, data saturation is not determined by recruitment itself, but is constrained by the overall study timeline. Given the established thresholds in other forms of qualitative research [67], we expect the targeted sample size of 105 participants to be sufficient to provide an adequate volume of qualitative data and reach a satisfactory measure of saturation.

## Ethics and dissemination

### Harms and risks management

#### Regulatory compliance and reporting.

According to Human Research Ordinance article 7 the risk category of this study is “A”. Study procedures and reporting standards are implemented and will be handled in accordance with Human Research Ordinance articles 12, 20, 21 and with the Human Research Act article 15. The appropriate bodies and individuals of responsibility will be contacted as outlined in the articles above. Should circumstances arise which could jeopardize participants’ health or safety or lead to a disproportionate relationship between risks and benefits of participants, all required measures will be taken without delay to ensure protection. The project leader and sponsor will be notified within 24 hours if such measures must be taken during the study and the ethics committee within 7 days of these measures and their circumstances.

#### Monitoring and participant safety measures.

The study team collects feedback (including adverse events and unintended effects of the intervention of trial conduct) communicated by phone, email, or through the application as part of the monitoring and tracking processes each workday. Should participants experience relevant distress or exacerbation of symptoms during any module, they are advised to pause that module and contact their primary care physician or specialized PCC clinic for further evaluation. If participants report serious or ongoing psychological distress, the study team provides a list of mental health resources, including contact information for crisis helplines, (psychosomatic) specialists and emergency clinics. These procedures are detailed in our standard operating procedures to ensure timely referral and participant safety.

#### Potential risks of study participation.

Participants may encounter the following risks:

- Increased cognitive load and distress: Exposure to PCC-related information may heighten cognitive strain or worrisome thoughts.
- Unsupervised execution of exercises: While exercises (e.g., breathing techniques) are presented verbally or visually, participants perform them without direct supervision from the study team. They are instructed to practice safely and responsibly and informed that exercises can be paused or omitted at any time if necessary.

### Protocol amendments

All amendments are communicated to the relevant ethics committee. Currently, there are no plans to communicate amendments beyond this submission process. The following amendments have been approved:

- 1^st^ Amendment: submitted March 18, 2024, and approved March 22, 2024
- 2^nd^ Amendment: submitted June 3, 2024, and approved June 18, 2024
- 3^rd^ Amendment: submitted August 30, 2024, and approved September 2, 2024
- 4^th^ Amendment: submitted December 20, 2024, and approved December 23, 2024
- 5^th^ Amendment: submitted March 10, 2025, and approved March 11, 2025

The complete ethics protocol with all amendments is available as supporting information (see S3 File).

### Access and security

The Docdok.health system operates in accordance with Swiss data protection laws and complies with the General Data Protection Regulation of the European Union.

All collected data will be encoded before analysis or, where appropriate, linked to a unique identifier (identical for BALCoS and DiLCoS). Access to data collection tools is restricted to authorized personnel and secured with password-protected logins. To add participants to the Docdok.health system, a phone number and email address are required for two-factor authentication and the following details are recorded: Salutation or title, first name, last name, gender, and date of birth. The following details are recorded on the Docdok.health platform: Salutation or title, first name, last name, gender, and date of birth. To ensure confidentiality, participant data is pseudonymized whenever possible, meaning that direct identifiers are replaced by a unique study identification. Participants can access their own information, which is also available only to authorized study team members.

#### Data availability and dissemination policy.

This manuscript describes the protocol of the DiLCoS study including research methods, data collection and management, as well as dissemination procedures. The study protocol does not report any findings of the described study, nor does this publication contain underlying participant-based data. As such, all data and information are in the manuscript and supporting files. The DiLCoS findings and results will be reported in peer-reviewed journals and key results are planned to be presented at conferences, symposia, lectures and presentations to the scientific community. Dissemination of results is additionally aligned within the Horizon Europe project to optimize impact. Authorship eligibility of this protocol publication is based on the guideline of the International Committee of Medical Journal Editors [68].

### Guidelines

We used the Standard Protocol Items: Recommendations for Interventional Trials [SPIRIT; 69] (see S1 File) and the Template for Intervention Description and Replication [TIDieR; 70] checklist (see S2 File) and guide in the preparation of this protocol.

### Artificial intelligence

We used artificial intelligence-based tools to support the preparation of this study protocol and manuscript. Specifically, we employed DeepL Translator [71] to check translations from German to English, ChatGPT (GPT-4, GPT-4o, and GPT-o3) [72] and Grammarly [73] to enhance language clarity and structure, and OpenEvidence [74] to assist with the literature search. We confirm that the contributions of AI were strictly in an assistive capacity and were not used for conceptual tasks. Human oversight was maintained at all times to ensure the accuracy of the content and address any ethical concerns.

## Supporting information

S1 File SPIRIT checklist. Filled standard protocol items: recommendations for interventional trials (SPIRIT) checklist.
(PDF)

S2 File TIDieR checklist. Filled template for intervention description and replication (TIDieR) checklist.
(PDF)

S3 File Ethics protocol. Most recent amended and approved ethics protocol.
(PDF)

S4 Fig Participant Timeline. Visualization participant timeline of the basel long COVID cohort study (BALCOS) and the digital long COVID study (DiLCoS).
(TIF)

## Abbreviations

ATS: ANOVA-type statistics
BALCoS: Basel Long COVID Cohort Study
BL: Baseline
CFS: 11-item Chalder Fatigue Scale
CNSVS: Central Nervous System Vital Signs
COVID-19: Coronavirus Disease 2019
DiLCoS: Digital Long COVID Study
EMA: Ecological Momentary Assessment
FMI: Fraction of missing information
GAD-7: Generalized Anxiety Disorder Questionnaire
GEE: Generalized Estimating Equations
ISI: Insomnia Severity Index
IPTW: Inverse-probability-of-treatment weights
LMM: Linear mixed model
MAR: Missing at Random
MICE: Multiple imputation by chained equations
PCC: Post-COVID-19 Condition
PHQ-8: Patient Health Questionnaire Depression Scale
PHQ-15: Patient Health Questionnaire Somatic Symptom Severity Scale
PSS-10: 10-item Perceived Stress Scale
QOL-8: Quality of Life: EuroHIS Quality of Life-8
RS-11: 11-item Resilience Scale
RTE: Relative-treatment effect
SARS-CoV-2: Severe Acute Respiratory Syndrome Coronavirus Type 2
SD: Standard Deviation
SMD: Standardized mean differences
T1: Post-Intervention Timepoint, 3 months after Baseline
T2: Post-Intervention Timepoint, 6 months after Baseline
T3: Post-Intervention Timepoint, 12 months after Baseline
TIDieR: Tempale for Intervention Description and Replication
UHB: University Hospital Basel
WHODAS 2.0: World Health Organization Disability Assessment 2.0
VAS: Visual Analogue Scales.

## References

[1] Al-Aly Z, Davis H, McCorkell L, Soares L, Wulf-Hanson S, Iwasaki A. Long COVID science, research and policy. Nature Medicine. 2024;30(8):2148–64. 10.1038/s41591-024-03173-6 39122965

[2] Thompson EJ, Williams DM, Walker AJ, Mitchell RE, Niedzwiedz CL, Yang TC, et al. Long COVID burden and risk factors in 10 UK longitudinal studies and electronic health records. Nat Commun. 2022;13(1):3528. doi: 10.1038/s41467-022-30836-0 35764621 PMC9240035

[3] Allwang C, Junne F. Post-Viral Phenomena after SARS-Cov-2: Implications for Psychosocial Care. Psychother Psychosom Med Psychol. 2021;71(12):487–8. doi: 10.1055/a-1645-4665 34872152

[4] Munblit D, O’Hara ME, Akrami A, Perego E, Olliaro P, Needham DM. Long COVID: aiming for a consensus. Lancet Respir Med. 2022;10(7):632–4. doi: 10.1016/S2213-2600(22)00135-7 35525253 PMC9067938

[5] Hastie CE, Lowe DJ, McAuley A, Winter AJ, Mills NL, Black C, et al. Outcomes among confirmed cases and a matched comparison group in the Long-COVID in Scotland study. Nat Commun. 2022;13(1):5663. doi: 10.1038/s41467-022-33415-5 36224173 PMC9556711

[6] Rochmawati E, Iskandar AC, Kamilah F. Persistent symptoms among post-COVID-19 survivors: A systematic review and meta-analysis. J Clin Nurs. 2024;33(1). 10.1111/jocn.16471 36426658

[7] Sugiyama A, Takafuta T, Sato T, Kitahara Y, Yoshinaga Y, Abe K, et al. Natural course of post-COVID symptoms in adults and children. Sci Rep. 2024;14(1):3884. doi: 10.1038/s41598-024-54397-y 38365846 PMC10873293

[8] Kenny G, Townsend L, Savinelli S, Mallon PWG. Long COVID: Clinical characteristics, proposed pathogenesis and potential therapeutic targets. Front Mol Biosci. 2023;10:1157651. doi: 10.3389/fmolb.2023.1157651 37179568 PMC10171433

[9] Hernández Ortiz OH, Naranjo Ramírez AF, Sierra Ramírez A, Restrepo Arias M, Betancourt Rodriguez N, Molina Saldarriaga FJ, et al. Post-COVID-19 syndrome: When an acute infection causes a chronic illness. Acta Colombiana de Cuidado Intensivo. 2024;24(4):387–97. doi: 10.1016/j.acci.2024.05.001

[10] Engelmann P, Reinke M, Stein C, Salzmann S, Löwe B, Toussaint A, et al. Psychological factors associated with Long COVID: a systematic review and meta-analysis. EClinicalMedicine. 2024;74:102756. doi: 10.1016/j.eclinm.2024.102756 39764180 PMC11701445

[11] Matta J, Wiernik E, Robineau O, Carrat F, Touvier M, Severi G, et al. Association of Self-reported COVID-19 Infection and SARS-CoV-2 Serology Test Results With Persistent Physical Symptoms Among French Adults During the COVID-19 Pandemic. JAMA Intern Med. 2022;182(1):19–25. doi: 10.1001/jamainternmed.2021.6454 34747982 PMC8576624

[12] Magnusson K, Turkiewicz A, Flottorp SA, Englund M. Prevalence of long COVID complaints in persons with and without COVID-19. Sci Rep. 2023;13(1). 10.1038/s41598-023-32636-y PMC10100609 37055494

[13] Schauenburg H. Post-COVID and ME/CFS – do we need new disease theories?. Zeitschrift für Psychosomatische Medizin und Psychotherapie. 2023;69(4):304–15. 37830884 10.13109/zptm.2023.69.oa7

[14] Norton A, Olliaro P, Sigfrid L, Carson G, Paparella G, Hastie C, et al. Long COVID: tackling a multifaceted condition requires a multidisciplinary approach. Lancet Infect Dis. 2021;21(5):601–2. doi: 10.1016/S1473-3099(21)00043-8 33548193 PMC7906694

[15] Mueller MR, Ganesh R, Hurt RT, Beckman TJ. Post-COVID Conditions. Mayo Clinic Proceedings. 2023;98(7):1071–8. 37419575 10.1016/j.mayocp.2023.04.007

[16] Nehme M, Diem L, Bassetti CLA, Guessous I. Swiss recommendations for the diagnosis, management and follow-up of post-COVID condition in primary care medicine (2023). Swiss Med Wkly. 2023;153:3468. doi: 10.57187/s.3468 37769668

[17] COVID-19 rapid guideline: managing the longterm effects of COVID-19. 2022. https://www.nice.org.uk/guidance/ng188/resources/covid19-rapid-guideline-managing-the-longterm-effects-of-covid19-pdf-51035515742

[18] POST-COVID RECOMMENDATIONS FOR PRIMARY CARE PHYSICIANS. 2023. https://www.rafael-postcovid.ch/sites/default/files/inline-files/HUG-POSTCOVID-EN%20-A4-FINAL_PROD.pdf

[19] Dalbosco-Salas M, Torres-Castro R, Rojas Leyton A, Morales Zapata F, Henríquez Salazar E, Espinoza Bastías G, et al. Effectiveness of a Primary Care Telerehabilitation Program for Post-COVID-19 Patients: A Feasibility Study. J Clin Med. 2021;10(19):4428. doi: 10.3390/jcm10194428 34640447 PMC8509356

[20] Kortianou E, Tsimouris D, Mavronasou A, Lekkas S, Kazatzis N, Apostolara Z, et al. Application of a home-based exercise program combined with tele-rehabilitation in previously hospitalized patients with COVID-19: A feasibility, single-cohort interventional study. Pneumon. 2022;35(2):1–10. doi: 10.18332/pne/146521

[21] Li J, Xia W, Zhan C, Liu S, Yin Z, Wang J, et al. A telerehabilitation programme in post-discharge COVID-19 patients (TERECO): a randomised controlled trial. Thorax. 2022;77(7):697–706. doi: 10.1136/thoraxjnl-2021-217382 34312316 PMC8318721

[22] Zeraatkar D, Ling M, Kirsh S, Jassal T, Shahab M, Movahed H, et al. Interventions for the management of long covid (post-covid condition): living systematic review. BMJ. 2024;387:e081318. doi: 10.1136/bmj-2024-081318 39603702 PMC11600537

[23] Schröder J, Bäuerle A, Jahre LM, Skoda E-M, Stettner M, Kleinschnitz C, et al. Acceptance, drivers, and barriers to use eHealth interventions in patients with post-COVID-19 syndrome for management of post-COVID-19 symptoms: a cross-sectional study. Ther Adv Neurol Disord. 2023;16:17562864231175730. doi: 10.1177/17562864231175730 37255668 PMC10225791

[24] Krotz A, Sosnowsky-Waschek N, Bechtel S, Neumann C, Lohkamp M, Kovacs G, et al. Reducing sick leave, improving work ability, and quality of life in patients with mild to moderate Long COVID through psychosocial, physiotherapeutic, and nutritive supportive digital intervention (MiLoCoDaS): study protocol for a randomized controlled trial. Trials. 2023;24(1):798. doi: 10.1186/s13063-023-07819-7 38066618 PMC10709981

[25] El-Toukhy S, Hegeman P, Zuckerman G, Anirban RD, Moses N, Troendle JF. A prospective natural history study of post acute sequalae of COVID-19 using digital wearables: Study protocol. Res Sq. 2023.

[26] Blanchard M, Backhaus L, Ming Azevedo P, Hügle T. An mHealth App for Fibromyalgia-like Post-COVID-19 Syndrome: Protocol for the Analysis of User Experience and Clinical Data. JMIR Res Protoc. 2022;11(2):e32193. doi: 10.2196/32193 34982039 PMC8820761

[27] Victoria LW, Oberlin LE, Ilieva IP, Jaywant A, Kanellopoulos D, Mercaldi C, et al. A digital intervention for cognitive deficits following COVID-19: a randomized clinical trial. Neuropsychopharmacology. 2024;50(2):472–9. doi: 10.1038/s41386-024-01995-z 39358543 PMC11631953

[28] Yang J, Li H, Zhao H, Xie Y, Li J, Wang M. Effectiveness of telerehabilitation in patients with post-COVID-19: a systematic review and meta-analysis of randomised controlled trials. BMJ Open. 2024;14(7):e074325. doi: 10.1136/bmjopen-2023-074325 38964791 PMC11227776

[29] Rinn R, Gao L, Schoeneich S, Dahmen A, Anand Kumar V, Becker P, et al. Digital Interventions for Treating Post-COVID or Long-COVID Symptoms: Scoping Review. J Med Internet Res. 2023;25:e45711. doi: 10.2196/45711 36943909 PMC10131666

[30] Harenwall S, Heywood-Everett S, Henderson R, Godsell S, Jordan S, Moore A, et al. Post-Covid-19 Syndrome: Improvements in Health-Related Quality of Life Following Psychology-Led Interdisciplinary Virtual Rehabilitation. J Prim Care Community Health. 2021;12:21501319211067674. doi: 10.1177/21501319211067674 34939506 PMC8721676

[31] Kumar N, Khunger M, Gupta A, Garg N. A content analysis of smartphone-based applications for hypertension management. J Am Soc Hypertens. 2015;9(2):130–6. doi: 10.1016/j.jash.2014.12.001 25660364

[32] Background: Horizon Europe Long COVID Project. 2022. https://longcovidproject.eu/background/

[33] Faul F, Erdfelder E, Lang A-G, Buchner A. G*Power 3: a flexible statistical power analysis program for the social, behavioral, and biomedical sciences. Behav Res Methods. 2007;39(2):175–91. doi: 10.3758/bf03193146 17695343

[34] Torres G, Gradidge PJ. The quality and pattern of rehabilitation interventions prescribed for post-COVID-19 infection patients: A systematic review and meta-analysis. Prev Med Rep. 2023;35:102395. doi: 10.1016/j.pmedr.2023.102395 37705882 PMC10495653

[35] Rohner S, Schnepper R, Meinlschmidt G, Schaefert R, Mayr M, Bopp K, et al. The Basel Long COVID Cohort Study (BALCoS): protocol of a prospective cohort study. medRxiv. 2024. doi: 2024.10.29.24316282 10.1136/bmjopen-2024-093981 PMC12248209 40645616

[36] WHO. A clinical case definition of post COVID-19 condition by a Delphi consensus. Geneva: World Health Organization. 2021.

[37] Docdok.health. 2022. https://www.docdok.health

[38] Rohner S, Schnepper R, Meinlschmidt G, Schaefert R, Mayr M, Bopp K, et al. Basel Long COVID Cohort Study (BALCoS): protocol of a prospective cohort study. BMJ Open. 2025;15(7):e093981. doi: 10.1136/bmjopen-2024-093981 40645616 PMC12248209

[39] Üstün TB, Kostanjsek N, Chatterji S, Rehm J. Measuring health and disability: Manual for WHO disability assessment schedule WHODAS 2.0. World Health Organization. 2010. 10.2471/BLT.09.067231 PMC2971503 21076562

[40] Carlozzi NE, Kratz AL, Downing NR, Goodnight S, Miner JA, Migliore N. Validity of the 12-item World Health Organization Disability Assessment Schedule 2.0 (WHODAS 2.0) in individuals with Huntington disease (HD). Qual Life Res. 2015;24(8):1963–71. 25636661 10.1007/s11136-015-0930-x PMC4497948

[41] Ferro MA, Dol M, Basque D, Elgie M. Validating the 12-item proxy-administered World Health Organization Disability Assessment Schedule (WHODAS) 2.0 in young children with chronic physical illness in Canada. Disabil Rehabil. 2023;45(19):3135–42. doi: 10.1080/09638288.2022.2118868 36093897

[42] Yang L, Wang D, Li X, Yuan H, Fang H, Guo X. Comparison of the responsiveness of the WOMAC and the 12-item WHODAS 2.0 in patients with Kashin-Beck disease. BMC Musculoskelet Disord. 2020;21(1):188. doi: 10.1186/s12891-020-03210-8 32213176 PMC7098162

[43] Brooke J. SUS: A “quick and dirty” Usability Scale. Usability Evaluation in Industry. 1996.

[44] Sauro J, Lewis JR. Quantifying the user experience: Practical statistics for user research. Morgan Kaufmann. 2016.

[45] Mayring P. Qualitative inhaltsanalyse. Konstanz: UVK Univ.-Verl. 1994.

[46] Crichton N. Visual analogue scale (VAS). J Clin Nurs. 2001;10(5):706–6.

[47] Schmidt S, Mühlan H, Power M. The EUROHIS-QOL 8-item index: psychometric results of a cross-cultural field study. Eur J Public Health. 2006;16(4):420–8. doi: 10.1093/eurpub/cki155 16141303

[48] Kroenke K, Spitzer RL, Williams JBW. The PHQ-15: validity of a new measure for evaluating the severity of somatic symptoms. Psychosom Med. 2002;64(2):258–66. doi: 10.1097/00006842-200203000-00008 11914441

[49] Toussaint A, Murray AM, Voigt K, Herzog A, Gierk B, Kroenke K, et al. Development and Validation of the Somatic Symptom Disorder-B Criteria Scale (SSD-12). Psychosom Med. 2016;78(1):5–12. doi: 10.1097/PSY.0000000000000240 26461855

[50] Kroenke K, Strine TW, Spitzer RL, Williams JBW, Berry JT, Mokdad AH. The PHQ-8 as a measure of current depression in the general population. J Affect Disord. 2009;114(1–3):163–73. doi: 10.1016/j.jad.2008.06.026 18752852

[51] Williams N. The GAD-7 questionnaire. Occup Med. 2014;64(3):224.

[52] Kocalevent R-D, Zenger M, Heinen I, Dwinger S, Decker O, Brähler E. Resilience in the General Population: Standardization of the Resilience Scale (RS-11). PLoS One. 2015;10(11):e0140322. doi: 10.1371/journal.pone.0140322 26523927 PMC4629910

[53] Morin CM. Insomnia severity index. 1993.

[54] Jackson C. The Chalder Fatigue Scale (CFQ 11). Occup Med (Lond). 2015;65(1):86. doi: 10.1093/occmed/kqu168 25559796

[55] Cohen S, Kamarck T, Mermelstein R. A global measure of perceived stress. J Health Soc Behav. 1983;24(4):385–96. doi: 10.2307/2136404 6668417

[56] Young QR, Ignaszewski A, Fofonoff D, Kaan A. Brief screen to identify 5 of the most common forms of psychosocial distress in cardiac patients: validation of the screening tool for psychological distress. J Cardiovasc Nurs. 2007;22(6). 10.1097/01.JCN.0000297383.29250.14 18090195

[57] Herman M, Gudex C, Lloyd A, Janssen M, Kind P, Parkin D. Development and preliminary testing of the new five-level version of EQ-5D (EQ-5D-5L). Quality of Life Research: An International Journal of Quality of Life Aspects of Treatment, Care and Rehabilitation. 2011;20(10). 10.1007/s11136-011-9903-x PMC3220807 21479777

[58] Ludwig K, Graf von der Schulenburg JM, Greiner W. German Value Set for the EQ-5D-5L. Pharmacoeconomics. 2018;36(6). 10.1007/s40273-018-0615-8 PMC5954069 29460066

[59] Meier BP, Konrath S, Fetterman AK, Dillard AJ, James C, Weinstein E. Development and validation of the single-item mindfulness scale (SIMS). J Pers Assess. 2022;105(6). 10.1080/00223891.2022.2152348 36480592

[60] Zhang JW, Howell RT, Chen S, Goold AR, Bilgin B, Chai WJ, et al. “I have high self-compassion”: A face-valid single-item self-compassion scale for resource-limited research contexts. Clin Psychol Psychother. 2022;29(4):1463–74. doi: 10.1002/cpp.2714 35083797

[61] Koufaki P, Mercer TH, Naish PF. Effects of exercise training on aerobic and functional capacity of end-stage renal disease patients. Clin Physiol Funct Imaging. 2002;22(2):115–24. doi: 10.1046/j.1365-2281.2002.00405.x 12005153

[62] American Society for Surgery of the Hand. The Hand: Examination and Diagnosis. 2nd ed. Edinburgh: Churchill Livingstone. 1983.

[63] Butland RJ, Pang J, Gross ER, Woodcock AA, Geddes DM. Two-, six-, and 12-minute walking tests in respiratory disease. Br Med J (Clin Res Ed). 1982;284(6329):1607–8. doi: 10.1136/bmj.284.6329.1607 6805625 PMC1498516

[64] CNS Vital Signs. https://www.cnsvs.com/index.html

[65] Malmivaara A. Benchmarking Controlled Trial--a novel concept covering all observational effectiveness studies. Ann Med. 2015;47(4):332–40. doi: 10.3109/07853890.2015.1027255 25965700 PMC4673508

[66] Saldaña J. The coding manual for qualitative researchers. 2021.

[67] Hennink M, Kaiser BN. Sample sizes for saturation in qualitative research: A systematic review of empirical tests. Soc Sci Med. 2022;292:114523. doi: 10.1016/j.socscimed.2021.114523 34785096

[68] ICoMJ. Recommendations for the conduct, reporting, editing, and publication of scholarly work in medical journals. 2024. https://www.icmje.org/recommendations/2024 25558501

[69] Chan A-W, Tetzlaff JM, Altman DG, Laupacis A, Gøtzsche PC, Krleža-Jerić K, et al. SPIRIT 2013 statement: defining standard protocol items for clinical trials. Ann Intern Med. 2013;158(3):200–7. doi: 10.7326/0003-4819-158-3-201302050-00583 23295957 PMC5114123

[70] Hoffmann TC, Glasziou PP, Boutron I, Milne R, Perera R, Moher D, et al. Better reporting of interventions: template for intervention description and replication (TIDieR) checklist and guide. BMJ. 2014;348:g1687. doi: 10.1136/bmj.g1687 24609605

[71] DeepL SE. DeepL - AI-assisted translations. 2017. https://www.deepl.com/de/translator

[72] OpenAI. ChatGPT 2024. https://openai.com/chatgpt

[73] Grammarly Inc. Grammarly - AI writing assistance. 2009. https://www.grammarly.com/

[74] OpenEvidence. OpenEvidence (Version 2.3). Cambridge (MA): OpenEvidence Inc. 2025. https://www.openevidence.com/

