# Supplementary material for: Protocol of the digital long COVID study: A single-center, registry-based, feasibility and clinical evaluation study to investigate a 12-week digital intervention program for people affected by post-COVID-19 condition
Source: PLoS One. 2026 Jan 20;21(1):e0340385. doi: 10.1371/journal.pone.0340385 (PMC12818690; doi:10.1371/journal.pone.0340385)
Supplement: S3 File — Most recent amended and approved ethics protocol. (PDF) [file pone.0340385.s003.pdf]

Titel Deutsch
BALCoS – Basler Long COVID Kohortenstudie

English Title
BALCoS – Basel Long COVID Cohort Study

Research legislation:	Ordinance on human research with the exception of Clinical trials (HRO; Federal Office of Public Health, 2022).
Type of Research Project:	Research project involving human subjects
Risk Categorisation:	Risk category A acc. to ordinance HRO Art.7

Project leader:
Prof. Dr. rer. nat. Gunther Meinlschmidt, Research Director
Department of Psychosomatic Medicine
University Hospital Basel, Hebelstrasse 2, CH-4031 Basel
Email: gunther.meinlschmidt@unibas.ch

Co-project leaders:
Dr. Andrea Meienberg, Senior Physician
Outpatient Medical Clinic
University Hospital Basel, Petersgraben 4, CH-4031 Basel
Email: andrea.meienberg@usb.ch

Dr. Katrin Bopp, Senior Physician
Outpatient Medical Clinic
University Hospital Basel, Petersgraben 4, CH-4031 Basel
Email: katrin.bopp@usb.ch

Sponsor:
Prof. Dr. med. Rainer Schaefert, Chairman
Department of Psychosomatic Medicine
University Hospital Basel, Hebelstrasse 2, CH-4031 Basel
Email: rainer.schaefert@usb.ch

Co-sponsor:
PD Dr. Michael Mayr, Co-Chief Physician
Outpatient Medical Clinic
University Hospital Basel, Petersgraben 4, CH-4031 Basel
Email: michael.mayr@usb.ch

TABLE OF CONTENTS

TABLE OF CONTENTS 3
GLOSSARY OF ABBREVATIONS 4
1 BACKGROUND AND PROJECT RATIONALE 5
2 PROJECT OBJECTIVES AND DESIGN 8
2.1 Hypothesis and primary objective 8
2.2 Primary and secondary endpoints 9
2.3 Project design 9
3 PROJECT POPULATION AND STUDY PROCEDURES 11
3.1 Project population, inclusion and exclusion criteria 11
3.2 Recruitment, screening and informed consent procedure 11
3.3 Study procedures 12
3.4 Withdrawal and discontinuation 18
4 STATISTICS AND METHODOLOGY 18
4.1. Statistical analysis plan 18
4.2. Handling of missing data 19
5 REGULATORY ASPECTS AND SAFETY 20
5.1 Local regulations / Declaration of Helsinki 20
5.2 Notification of safety and protective measures (HRA Art. 15, HRO Art. 20) 20
5.3 Serious events (HRO Art. 21) 20
5.4 Procedure for investigations involving radiation sources 20
5.5 Amendments 20
5.6 End of project 20
5.7 Insurance 20
6 FURTHER ASPECTS 21
6.1 Overall ethical considerations 21
6.2 Risk-Benefit Assessment 21
6.3 Rationale for the inclusion of vulnerable participants 22
7 QUALITY CONTROL AND DATA PROTECTION 22
7.1 Quality measures 22
7.2 Data recording and source data 22
7.3 Overview of the DocDok System 23
7.4 Confidentiality and coding 23
7.5 Retention and destruction of project data and biological material 24
8 FUNDING / PUBLICATION / DECLARATION OF INTEREST 24
9 REFERENCES 26
APPENDIX 1: SCHEDULE OF ASSESSMENTS 32
APPENDIX 2: ADDITIONAL BLOOD BIOSPECIMENS TO BE SHARED WITH CONSORTIUM PARTNERS 33
APPENDIX 3: SCREENSHOTS FROM THE SMARTPHHONE APPLICATION AND THE PLATFORM 34

GLOSSARY OF ABBREVATIONS

ANOVA	*Analysis of variance*
BALCoS	*Basel Long COVID Cohort Study*
BASEC	*Business Administration System for Ethical Committees*
BL	*Baseline*
CFS	*Chalder Fatigue Scale*
CHF	*Swiss Francs*
COVID	*Coronavirus disease*
CNSVS	*Central Nervous System Vital Signs*
CPET	*Cardiopulmonary Exercise Testing*
CR	*Category-Ratio*
CRF	*Case report form*
eCRF	*Electronic case report form*
DNA	*Deoxyribonucleic acid*
DiLCoS	*Digital Long COVID Study*
EDTA	*Ethylenediaminetetraacetic acid*
EMA	*Ecological momentary assessment*
EKNZ	*Ethikkommission Nordwest- und Zentralschweiz*
EU	*European Union*
FOPH	*Federal Office of Public Health*
GAD	*General Anxiety Disorder*
GDPR	*General Data Protection Regulation*
GLM	*General linear model*
ISI	*Insomnia Severity Index*
HRA	*Human Research Act*
HRO	*Human Research Ordinance*
PCC	*Post COVID-19 condition*
PHQ	*Patient Health Questionnaire*
QOL	*Quality of life*
PSS	*Perceived Stress Scale*
RNA	*Ribonucleic acid*
RS	*Resilience Scale*
SARS-CoV-2	*Severe acute respiratory syndrome coronavirus 2*
SERI	*Swiss State Secretariat for Education, Research and Innovation*
SOP	*Standard operating procedure*
SSD	*Somatic Symptom Disorder*
STS	*Sit-to-tand*
SUS	*System Usability Scale*
UHB	*University Hospital Basel*
VAS	*Visual Analogue Scale*
WHO	*World Health Organization*
WHODAS	*World Health Organization Disability Assessment Schedule*

1 BACKGROUND AND PROJECT RATIONALE

The COVID-19 pandemic & post COVID-19 condition

Since early 2020, the coronavirus disease (COVID-19), caused by the severe acute respiratory syndrome coronavirus 2 (SARS-CoV-2) has led to a global pandemic. During the past years, research has advanced knowledge on the symptoms, treatment, and prevention of COVID-19 and vaccines have been developed. As the pandemic progressed, it became apparent that the disease does not only cause acute symptoms but can also lead to long-term complaints (sometimes termed "long COVID", in the following referred to as "post COVID-19 condition (PCC)" according to the definition of the World Health Organization (WHO)). A meta-analysis detected 55 different symptoms, signs, and abnormal laboratory parameters related to SARS-CoV-2 infection. Most of them were similar to symptoms observed during the acute phase of COVID-19, with fatigue being the most common symptom (Lopez-Leon et al., 2021).

In October 2021, an official WHO definition of PCC has been released, defining the condition as symptoms lasting for at least two months, usually within three months from the onset of COVID-19 that cannot be explained by an alternative diagnosis. Common symptoms include fatigue, shortness of breath, and cognitive dysfunction, and have a negative impact on daily life (WHO, 2021).

Relevance to public health

Until today, the initially described complaints of fatigue, shortness of breath, and cognitive dysfunction are still reported as the most frequent symptoms of PCC (Soriano et al., 2021). A recent modelling of the WHO estimated that around 17 million Europeans experienced PCC symptoms during the first two years of the pandemic (Wise, 2022). In a Dutch cohort study, around one in eight patients with COVID-19 experienced PCC symptoms (Ballering et al., 2022). In a Swiss cohort study that was conducted during the first year of the pandemic, even 26% of enrolled participants stated that they did not fully recover 6-8 months after being diagnosed with COVID-19 (Menges et al., 2021). A UK study reported that for PCC the mean duration of illness in a large population sample was 7.2 months (Ziauddeen et al., 2022). Almost half of the Swiss population has been tested positive for SARS-CoV-2 at least once as of October 2022 (Federal Office of Public Health, 2022), which underscores the high relevance of better understanding and treating PCC also from a Swiss perspective. In sum, PCC represents a major challenge for societies and health care systems due to high individual suffering, sickness and productivity losses, large health care utilization, as well as high direct and indirect health care costs.

Current state of knowledge

PCC is heterogeneous not only with regard to its manifestations, but also with regard to prevalence, progression, and duration (Nittas et al., 2021). Two different pathophysiological entities are discussed: on the one hand, patients with an initially severe course with tangible organ damage, and on the other hand, patients who have experienced an initially asymptomatic or mild course of the acute SARS-CoV-2 disease with post covid complaints whose symptoms (fatigue, exercise intolerance, shortness of breath) cannot yet be satisfactorily explained.

Multi-organ sequelae have been described, including hematological sequelae (e.g., thromboembolic events), cardiovascular sequelae (e.g., palpitations), neuropsychiatric sequelae (e.g., migraines), renal sequelae (e.g., following severe acute kidney injury during the acute phase of the disease), endocrine sequelae (e.g., type 1 diabetes), and dermatological sequelae (e.g., hair loss). The clinical, serological, imaging, and epidemiologic features of COVID-19 and PCC need to be identified and better characterized to increase understanding of its pathophysiology and course (Nalbandian et al., 2021). In the field of immunology and immunopathology, different hypotheses have been proposed regarding the etiopathology of PCC. These include amongst others chronic inflammation, autoimmune reactions after acute viral infection, dysbiosis of the

microbiome or the virome, and unremitting tissue damage. It has been suggested that immune stimulation might be driven by the persistence of the virus or viral antigens. PCC symptoms might further be mediated by the presence of autoantibodies (Merad et al., 2022).

Even though some risk factors of PCC could be identified, e.g. female gender, socioeconomic deprivation, smoking, obesity, and comorbid diseases, the etiology of PCC is still widely unclear (Subramanian et al., 2022). Due to the insufficient understanding of the mechanisms that underlie the development of PCC, treatment options are currently limited, and focus on alleviating the most burdening symptoms. This insufficient understanding potentially contributes that those affected by PCC may experience feelings of helplessness, frustration, being misunderstood, worry due to the uncertain course of the disease, and a sense of losing control of their lives (Loft et al., 2022). A better understanding of the pathogenesis as well as possible risk and protective factors of PCC would help improving health care and support of people suffering from PCC (Crook et al., 2021).

DiLCoS (Digital Long COVID Study) Substudy

Current recommendations for treatment of PCC

In public healthcare, the management and treatment of PCC presents a significant challenge (O'Hare et al., 2022; Peiris et al., 2023; Prashar, 2023). This challenge is being increasingly addressed through innovative approaches, particularly digital interventions and digital therapeutics (Blanchard et al., 2022; El-Toukhy et al., 2023; Krotz et al., 2023; Schröder et al., 2023)

Current State of Offline/Face-To-Face Treatment of PCC

Traditional management of PCC involves a multidisciplinary approach, integrating various medical specialties. Treatment guidelines vary by country, reflecting diverse healthcare systems and epidemiological perspectives. For instance, Switzerland's guidelines emphasize a symptom-centered approach, incorporating rehabilitation and psychological support (Nehme et al., 2023; Rafael Post-Covid Platform, 2023). The UK's National Institute for Health and Care Excellence (NICE) guideline suggests a comprehensive assessment followed by a personalized care plan (NICE, 2022). These guidelines underline the importance of addressing the multifactorial nature of PCC, yet they also highlight the limitations of traditional treatment modalities, such as resource constraints and accessibility issues.

Digital interventions and their application to chronic conditions

Digital interventions, encompassing digital therapeutics, represent an emerging field in healthcare, offering remote, scalable, and personalized treatment options. Defined as evidence-based therapeutic interventions driven by high quality software programs to prevent, manage, or treat a medical disorder or disease (Digital Therapeutics Alliance, 2019), these interventions are particularly relevant for chronic conditions. They may offer continuous monitoring, real-time feedback, and adaptive interventions, tailored to individual patient needs and conditions. This approach is revolutionizing chronic disease management, including diabetes, cardiovascular diseases, and mental health disorders, by enhancing patient engagement, treatment adherence, and overall health outcomes (Cuijpers et al., 2022; Dang et al., 2021; Wongvibulsin et al., 2021).

State of knowledge regarding digital interventions for PCC

The application of digital interventions in managing PCC is an emerging field. Key characteristics of these interventions include remote monitoring, symptom tracking, and personalized rehabilitation and symptom management programs. Several smartphone applications and digital platforms have been developed, aiming to address the broad spectrum of PCC symptoms. Rinn et al. (2023) discuss in their scoping review existing evidence-based digital interventions for symptom management in the context of PCC. They report that in most included studies breathing exercises, stretching, and similar forms of interventions improve physical health. With regard to mental health improvements, they noted that some studies show effects for psychological interventions, with one included study (Harenwall et al. (2021) reporting 37% of participants to have improved levels of anxiety and depression following intervention. They report that

information about sleep, stress management, and energy conservation as part of a larger rehabilitation courses can be an effective intervention (Harenwall et al., 2021). Rinn and colleagues report that some studies employed a multidisciplinary approach by encompassing both physical and psychological interventions (e.g., Harenwall et al., 2021; Kortianou et al., 2022). These results suggest that future interventions might pursue a mixed approach to better address people's needs and optimize benefits for participants. Based on the current state-of-knowledge, the DiLCoS substudy aims to fill this research gap by incorporating physical elements (in the form of breathing exercises, relief positions and relaxation techniques), psychoeducational elements (e.g., information about fatigue, neurocognitive symptoms, media consumption, healthy diet), self-monitoring elements (activity planning, energy management), and psychological elements (e.g., guided exercises to improve acceptance of current situation, cognitive distancing from negative thoughts, imagination techniques for pain management). Furthermore, Rinn et al. (2023) conclude that there is a general lack of theory-based studies investigating the efficacy of digital tools.

To address this current lack of comprehensive digital interventions, the DiLCoS substudy is based on a bio-psycho-social approach, employing evidence-based techniques (e.g., progressive muscle relaxation; PMR), and addresses different aspects of PCC in comprehensive manner and from a broad multidisciplinary perspective (e.g., the collaboration with expert teams on the development and review of module content). A further limitation of current research raised by Harenwell et al. (2021) is limited completion rates of outcome measure assessments. This is addressed by continuously monitoring progress and participants' engagement via weekly assessments.

Rationale of the research project

Given the described knowledge gap, the high number of affected patients, and the burden of the disease worldwide, this research project aims to foster the understanding of potential underlying mechanisms and causes of PCC as well as the dynamics of the disease, potentially informing new preventive and interventive approaches. To do so, our registry-based cohort study, the Basel Long COVID Cohort Study (BALCoS) will focus on the patients' present health status, symptoms, the course of these symptoms, and potential mechanisms involved. As PCC is a diagnosis based on the exclusion of other conditions that can explain the symptoms, we will also collect data on diagnostic procedures and their outcomes. Furthermore, the project aims to investigate proposed mechanisms behind PCC, including a) autoimmunity, b) chronic inflammation, c) genetics, d) coagulation disorders, and e) psychosocial factors. At the University Hospital Basel (UHB), we will invite patients seeking help for PCC symptoms at the UHB medical outpatient clinic to participate in BALCoS. We will further invite patients treated for PCC in other consultations from the German-speaking parts of Switzerland and the neighbouring Germany. Patients enrolled in BALCoS will complete a set of assessments that include the collection of sociodemographic and clinical data, biomarkers, neurocognitive testing, psychometric questionnaires, and measures of physical performance, and provide consent that their data from routine clinical care can be used for the study. As in this registry-based cohort study no intervention besides standard care including individual remedies is given to patients, no radiation is used, and no vulnerable group is recruited, the risk category of the proposed study is “A” according to ordinance HRO Art. 7.

DiLCoS Substudy

Considering the complexity and variability of PCC symptoms, a study evaluating the feasibility and proof-of-concept of exercises delivered via a smartphone application (referred to hereinafter as digital intervention) is highly warranted. Such a digital intervention has the potential to address several unmet needs in PCC management. It can provide personalized support plans and adaptive strategies based on patient feedback. The integration of both passive (e.g., psychoeducational content) and active (e.g., guided exercises) elements can enhance the understanding and management of this condition. Importantly, a smartphone application that covers a broad spectrum of symptoms and adapts to patients' changing needs could significantly improve patient outcomes and quality of life, filling a critical gap in current treatment approaches.

DiLCoS is predicated on the recognition that PCC, with its complex array of symptoms, requires an interdisciplinary, multimodal treatment approach that extends beyond conventional medical interventions.

The rationale for DiLCoS is anchored in several key considerations:

- Comprehensiveness of the intervention: PCC presents a spectrum of symptoms including fatigue, dyspnea, and cognitive disturbances, which necessitate a comprehensive treatment approach (Mueller et al., 2023; Norton et al., 2021). DiLCoS, through its series of modules, offers a holistic support framework that encompasses not just the physical symptoms but also the psychological and psychosocial aspects of PCC. This comprehensive approach is informed by the emerging evidence suggesting the effectiveness of multifaceted interventions (e.g., Dalbosco-Salas et al., 2021; Kortianou et al., 2022; Li et al., 2022).
- Digital format and accessibility: The digital format, specifically the smartphone application, ensures greater accessibility and convenience for patients. By providing resources through a smartphone application, DiLCoS ensures that support and guidance are readily available, overcoming geographical and physical barriers that often limit access to care. Especially considering that the smartphone application is equipped with a text-based chat function in order to communicate effectively and efficiently with the study team.
- Integration of passive and active elements: The incorporation of both passive (e.g., psychoeducational content) and active (e.g., exercises, self-reflection diaries) components in the smartphone application ensures a dynamic and interactive intervention. This dual approach is crucial in managing chronic symptoms, as it aids adapting to the patient's condition and promotes self-management (Kumar et al., 2015).
- Evidence-based content and structure: The content and structure of DiLCoS are informed by latest research and past successful digital interventions for PCC. Each module targets specific domains of PCC, based on evidence-based practices for symptom management and improvement (e.g., Dalbosco-Salas et al., 2021; Kortianou et al., 2022; Li et al., 2022).

In summary, the DiLCoS substudy aims to respond to the pressing need for innovative, accessible, and effective interventions for PCC. Its design in the form of a smartphone application, which integrates comprehensive care, digital accessibility, active and passive engagement, adaptability to patient needs, and evidence-based content, holds promise for significantly improving the management and quality of life of individuals living with PCC.

2 PROJECT OBJECTIVES AND DESIGN

2.1 Hypothesis and primary objective

As described above, the primary aim of BALCoS is to get a more thorough understanding of PCC, including symptoms and course, as well as underlying pathophysiological mechanisms. Therefore, we will conduct a single-center registry-based cohort study consisting of patients suffering from PCC. Within this cohort, we will look into clinical manifestations, risk and protective factors, and underlying mechanisms for the development and course of PCC. BALCoS is conducted in the context of a larger project carried out by an EU-wide consortium and will contribute data and biospecimens to this consortium.

Specifically, within the consortium, results from clinical and mechanistic projects shall together inform the scrutinization of molecular and physiological parameters and/or pathways to decipher the mechanisms underlying PCC and to develop tools and knowledge to support physicians in accurately managing PCC. Furthermore, data shall be used to inform a machine learning and AI-informed PCC Prediction Support tool for future use by clinicians to predict PCC and its possible clinical manifestations in patients and personalize their treatments.

DiLCoS Substudy

The aim of the substudy is to gather proof of concept regarding the effectiveness and feasibility of providing the digital intervention outlined above via a smartphone application. The substudy will explore effects on symptom management, user engagement, and satisfaction with the digital intervention. This will involve evaluating how frequently participants use the smartphone application, and their feedback on its usability and helpfulness. In addition, the feasibility of the intervention material will be assessed by user-provided feedback on the length and comprehensiveness of the content.

2.2 Primary and secondary endpoints

As this study is designed as a registry-based cohort study, there are no standard primary and secondary endpoints. Rather, measures from the fields of clinical medicine, virology, metabolism, and psychosomatics will be combined for a comprehensive understanding of PCC. These measures include sociodemographic information, clinical data (retrieved from regular care for PCC in the UHB medical outpatient clinic or, for patients from other consultations, from their doctor's reports and from an interview at baseline conducted by study personnel), biomarkers derived from blood samples, neurocognitive testing, psychometric questionnaires, and measures of physical performance. More detailed information about the interview process can be found in the Case Report Form. Baseline comparisons as well as the longitudinal course of PCC will be investigated to gain a better understanding of PCC. Please refer to "3.3 Study procedures" and appendix 1 for details and schedules of these measures.

DiLCoS Substudy
Primary Endpoint

The primary endpoint for DiLCoS will be the change in participants' functional capacity (T0 compared to T1), as measured by WHODAS 2.0 (12-item version; Üstün et al., 2010). This measure will provide a quantifiable assessment of the impact of the digital intervention on participants' daily functioning and disability levels.

Secondary Endpoints (see also Appendix 1)

Psychometric Questionnaire Data: Questionnaires employed within BALCoS to evaluate mental health status, quality of life and other psychosomatic factors will offer insights into the broader impact of the intervention and provide data for the longevity of possible effects emerging from DiLCoS.

Neurocognitive Testing: Neurocognitive function, which can be affected in PCC, will be assessed as a secondary endpoint. CNS Vital Signs (CNSVS, https://www.cnsvs.com/) will be assessed within BaLCoS at baseline and T3. This will help in evaluating any cognitive improvements as a result of the digital intervention.

Measures of Physical Performance: Physical performance metrics, measured at baseline and T3 within BaLCoS, assess improvements in physical health and capacity, which are critical components of overall recovery in PCC. Possible changes in physical performance metrics can provide insight into the effectiveness of exercises, especially physical elements, within DiLCoS.

2.3 Project design

This is a single-center, observational registry-based cohort study. The study site will be located at the medical outpatient clinic unit at the UHB, which has started a service specializing in PCC patients in 2021 due to the high demand in the population. This service collaborates with experts from numerous disciplines relevant for managing PCC patients, including e.g. cardiology, neurology, pneumology, otorhinolaryngology, psychosomatic medicine, and physiotherapy. All patients visiting the PCC speciality clinic at the UHB

i. will be invited to participate in BALCoS. To this end, they will also be asked to provide informed consent that
ii. data collected within BALCoS and
iii. data from their routine clinical care can be used for future research purposes. Further,
iv. patients will be asked to give consent that they can be recontacted for potential additional follow-up assessments

Please note that ii to iv are no prerequisite for participating in BALCoS and that i is no prerequisite for iii and iv.
We will further get in touch with physicians from other PCC consultations from the German-speaking parts of Switzerland and the neighbouring Germany and will ask them to advertise the study in their consultation.

BALCoS is primarily funded by the Swiss State Secretariat for Education, Research and Innovation (SERI, under funding number: 22.00094) in the context of a European Union (Horizon Europe) research consortium "Long Covid" (funding number: 101057553). Data collected within BALCoS will be analyzed on its own, but will also contribute to a geographically diverse pooled data set derived from studies involved in the above-mentioned EU/SERI funded consortium, i.e., biospecimens being sent and data being shared (refer to appendix 2 and Joint Controllership Agreement). Data from all the EU consortium (from The Netherlands, Finland, and Switzerland) shall be used to develop an AI-driven prediction tool for clinical decision support and shared decision-making (please also refer to the Joint Controllership Agreement).

Prospectively, approximately 6 months after data collection has started, new patients included in BALCoS are invited to participate in the DiLCoS substudy, a proof-of-feasibility substudy of a digital intervention with personalized, multimodal digital interventions. The initial protocol has been amended to comprise the DiLCoS substudy.

We intend to continue patient inclusion in BALCoS beyond the current SERI-based funding period, allowing further data analyses and a continuing scrutinizing of the above-mentioned research questions, including changes in PCC over time.

DiLCoS Substudy

The intervention substudy is designed as a single-arm, cohort-based proof-of-feasibility study and is part of the larger BALCoS study. The study team developed the material in six stages.

- Stage 1: The latest publications, evidence-based methods and techniques, and available information regarding a module (for description of modules see Point 3.3; Study Procedures) was consulted, selected, filtered and condensed into module scripts. These scripts outlined all elements of a module (introduction, exercises, psychoeducational content, conclusionary statement) and all members of the interdisciplinary study team were asked to provide feedback. For module 2 (healthy lifestyle), a member of the nutritional counselling team at UHB was asked to review the material. For module 3 (physical activity), the physical therapy team at UHB was asked to collaborate in the development of the script. The first stage concluded with agreement on the content of the module script within the study team and where applicable the expert teams.
- Stage 2: The final scripts were reworked into standardized instructions for digital implementation. The content of the scripts was allocated to the days of the corresponding module (see Figure 2). Pictures and illustrations under the Creative Commons Zero (https://creativecommons.org/publicdomain/zero/1.0/) license were used to accompany the written content. The instructions were again reviewed by study team members to ensure correct implementation of the content into digital elements (recordings and edits of interviews, animated videos and audio guides).
- Stage 3: Digital elements were produced, recorded, and edited. Afterwards, they were again reviewed by the team.
- Stage 4: The finished material as well as implementation instructions were uploaded to a cloud-based storage platform for implementation into the smartphone content system

application (provided by DocDok.health; See 7.3 Overview of DocDok System).
- Stage 5: Content implemented by DocDok.health was again reviewed by the study team.
- Stage 6: Content was approved and the final version of each module was uploaded to the smartphone content system application.

3 PROJECT POPULATION AND STUDY PROCEDURES

3.1 Project population, inclusion and exclusion criteria

Patients diagnosed with PCC or patients who subjectively attribute their symptoms to PCC will be included. For inclusion, signed informed consent is required. Following WHO guidelines, PCC will be defined as:

- History of confirmed or suspected SARS CoV-2 infection
- Symptoms usually start within 3 months from the onset of acute SARS CoV-2 infection with symptoms and effects that last for at least 2 months
- Symptoms cannot be explained by an alternative diagnosis

Exclusion criteria will be:

- Age <18 years
- Lack of consent to participate in the study
- Language barriers (lack of sufficient knowledge of German)
- Lack of general understanding of study procedures

DiLCoS Substudy

Participants enrolled in BALCoS who have completed the baseline measurements, but not yet T1 measurements, are asked to participate in the DiLCoS substudy. Due to a decrease in PCC referrals, patients who are further progressed in BALCoS (beyond T1) will also be invited to take part in the intervention. To evaluate their participation in the intervention, they will again complete the measures described in section below (with the last measure completed within 3 months before the start of the intervention being the new BL). Their refusal of participation in DiLCoS or withdrawal of consent during participation in DiLCoS does not affect their participation in BALCoS in any manner. There is one additonal inclusion criterion for the DiLCoS substudy, which is the possession of a smartphone with an operating system that is compatible with software comprising the intervention. For being included in the DiLCoS substudy, separate signed informed consent is required (see separate document).

3.2 Recruitment, screening, and informed consent procedure

Patients will be recruited at the medical outpatient clinic unit at the UHB from February 2023 on. From this ongoing recruitment, we expect to include at least N=120 patients within the first three years of BALCoS (first patient in February 2023, first patient out February 2024; last patient in March 2025, last patient out March 2026). Recruitment shall be ongoing beyond the SERI funding phase. Enrolment will be based on the inclusion criteria mentioned above and will be carried out by the study team which includes physicians and specialized study nurses. Any patient fulfilling inclusion criteria and lacking exclusion criteria will be asked to participate in the study. The study investigator (or their designee) will obtain written informed consent from all study participants. The investigators will explain to each patient the nature of the study, its purpose, the procedures involved, the expected duration, the potential risks and benefits, and any discomfort it may entail. Each patient will be informed that participation in the study is voluntary and that he/she may withdraw from the study at any time and that withdrawal of consent will not affect his/her subsequent medical assistance and treatment. Patients will be compensated for visits that exceed standard care (see 3.3 Study procedures). The study will also be advertised by flyers sent to patients who have an appointment at the PCC outpatient clinic at the UHB before the scheduled appointment (see separate document).

Since a drop in patients was observed from December 2023 onwards, we will also invite patients treated for PCC in other consultations as well as patients who participated in PCC studies in the past from the German-speaking parts of Switzerland, Germany, and Austria. We will do so by sending physicians flyers and posters with a QR-code leading to the flyer for external patients (see separate documents) that advertise both BALCoS and DiLCoS and will ask them to advertise the study in their consultation. We will also advertise the study in PCC patient networks, press releases, and by posters in public buildings and spaces. Further, the study will be advertised online (e.g., via advertisements on social media, Google, and native advertisements). Texts will be slightly adjusted due to space constraints for the different advertising materials. However, every ad format will always link to the (approved) study advertisement. See separate documents for advertising material. In order to participate, they are required to have a report from a doctor stating a suspected or confirmed diagnosis of PCC or attribute their symptoms subjectively to PCC.

DiLCoS Substudy

Recruitment for the DiLCoS substudy is connected to the recruitment for the BALCoS study. Once DiLCoS has started, recruitment will take place as long as recruitment for BALCoS is ongoing. Potential participants will firstly be presented with study information for BALCoS and secondly for DiLCoS and the study team will explain the nature, purpose, procedures involved, the expected duration, potential risks and benefits, and any discomfort it may entail for both research projects. A separate written consent for the DiLCoS substudy will be obtained. Consent and therefore participation in BALCoS is a requirement for participation in DiLCoS as outlined in section 3.1.

3.3 Study procedures

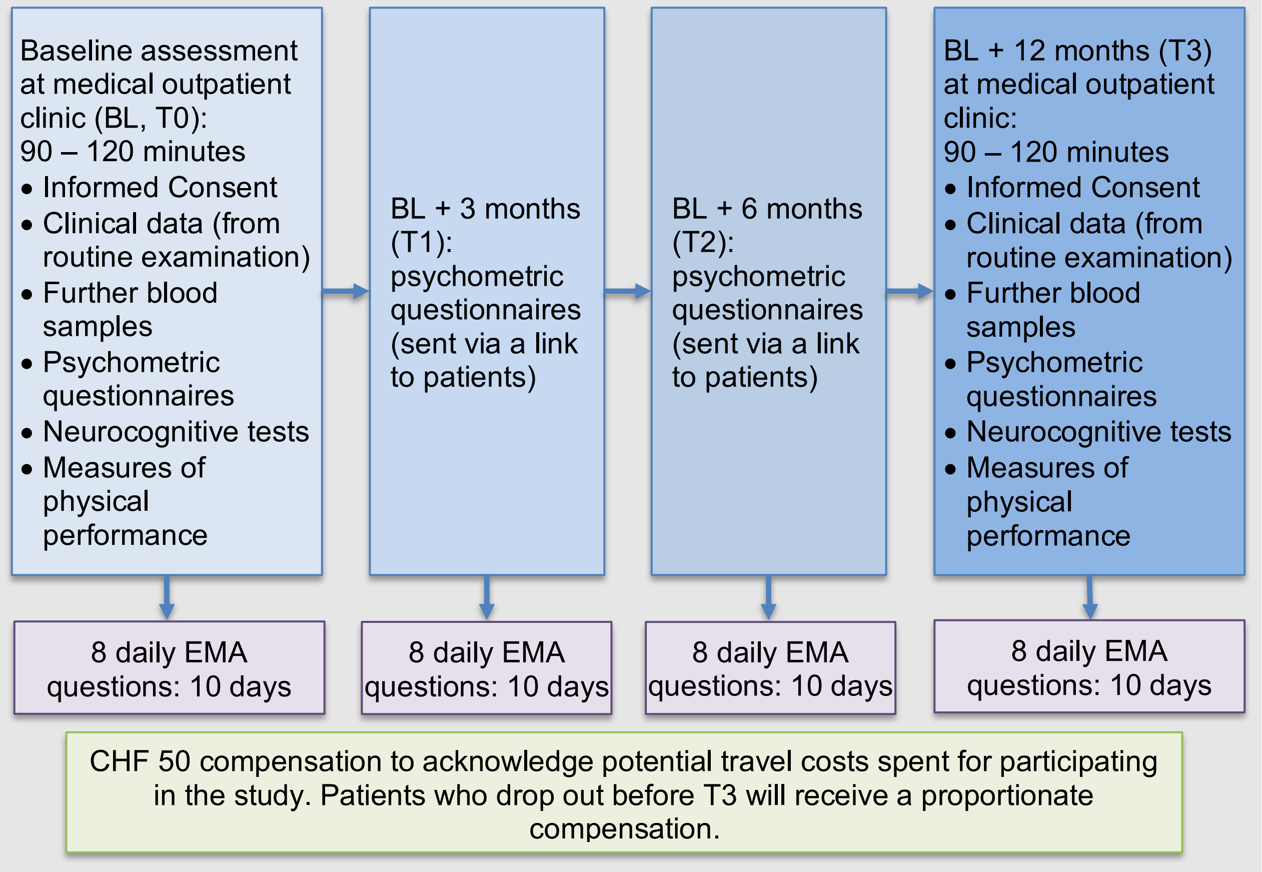

Figure 1. Study procedures. At Baseline (BL) and T3, psychometric measures will be completed via a tablet-based assessment whenever possible. Neurocognitive tests will be completed on a computer. If the duration of the BL assessment is too much of a strain for a patient to complete in one visit, a second visit on a separate day can be appointed, on which the neurocognitive test

battery and measures of physical performance will be assessed. If the respective consent was given, patients might be recontacted for additional assessments after T3. See appendix 1 for a summary table listing all project visits including relevant procedures, sampling, and timelines.

Detailed information on measures

Clinical Data:

Patients will be asked to give consent that all data from previous medical examinations that might be relevant to their PCC symptomatology as well as at intake will be included in the study. Further, they will be asked to give consent that data assessed in the study can be used to inform clinical routine care.

Blood Samples:

In addition to blood drawn during the routine clinical assessment, blood samples for further analyses will be drawn at BL and T3. Blood samples that are shipped to consortium partners are described in Appendix 2 and in separate SOP forms. With these samples, genome and HLA typing (plasma), COVID-19 antibody profiling (serum), lipidome (serum), autoantibody epitopes (serum), and citrated plasma for coagulation analyses will be collected and investigated (please note that due to limited resources, for some parameters only specimens from a subsample of the enrolled patients will be sent and analyzed). Patients will be given the option to decline genome analyses and still participate in the study. In addition to the shipped blood samples, blood samples for local analyses at the UHB will be drawn. These blood samples are

- 3 PAXGene Blood RNA Tubes (genetic analyses; 2.5ml each)
- 1 x 7.5 ml EDTA (autoantibodies; prepare plasma aliquots à 1ml, freeze the cell pellet (DNA sequencing))
- 1 x 7.5 ml serum tube (immunology and hematology; prepare serum aliquots à 1ml, discard the clotted blood))

The total amount of blood drawn for BALCoS will be 45 ml.
Patients from external consultations will only be asked for the samples described in Appendix 2 to be drawn (until the predefined numbers of samples from a maximum of *n*=120 patients are reached).

Neurocognitive Testing:

CNS Vital Signs (CNSVS, https://www.cnsvs.com/) is a computerized neurocognitive test battery that was developed as a routine clinical screening instrument. It consists of seven scientifically valid and reliable neuropsychological tests that normally require an interviewer. The tests are: verbal and visual memory, finger tapping, symbol digit coding, the Stroop Test, a test of shifting attention, and the continuous performance test. Completion of the tests takes 40-50 minutes. The psychometric properties of the CNSVS tests have been shown to be comparable to the interview-based conventional neuropsychological tests from which they are derived (Gualtieri & Johnson, 2006).

Psychometric questionnaires:

The *World Health Organization Disability Assessment Schedule (WHODAS 2.0)* assesses and classifies disability due to health problems during the past 4 weeks. This study will utilize the 12-item version (Üstün et al., 2010). An example item would be “In the past 4 weeks, how much difficulty did you have in walking a long distance such as a kilometer (or equivalent)?”. Items are answered on a 5-point scale ranging from 0 = “none” to 4 = “extreme or cannot do”.

Visual analogue scales (VAS): Patients will answer 4 questions on a VAS with a rating slider ranging from 0 to 10. The questions will inquire symptom intensity (10 = it can’t be worse), functional impairment (10 = can’t be worse), quality of life (10 = can’t be better), and work capacity (10 = full work capacity). For work capacity, patients will also indicate their current incapacity to work and highest incapacity to work in relation to PCC on a slider from 0% - 100%.

As a measure of life quality, the *EuroHIS Quality-of-Life-8* (QOL-8; Schmidt et al., 2005) will be used. This 8-item instrument assesses quality of life and perceived health during the past 4 weeks, e.g. by asking “Do you have enough energy for everyday life?”. Items are answered on a

5-point scale, with wording differing between questions (e.g., from “very dissatisfied” to “very satisfied”).

The S*omatic Symptom Disorder Questionnaire* (SSD-12; Toussaint et al., 2016) assesses psychological features of somatic disorders, e.g. catastrophizing thoughts and health anxiety, e.g. with the statement “I think that my physical symptoms are signs of a serious illness”. Items are answered on a 5-point scale ranging from 0 = “never” to 4 = “very often”.

The *Patient Health Questionnaire* (PHQ-15; Kroenke et al., 2002) assesses somatic symptom severity during the past 4 weeks. Patients are asked how impaired they felt by their symptoms, e.g. back pain. Items are answered on a 3-point scale ranging from 0 = “not bothered at all” to 2 = “bothered a lot”.

The 8-item *Patient Health Questionnaire Depression Scale* (PHQ-8; Kroenke et al., 2009) assesses the severity of depressive disorders. Patients are asked if in the past weeks, they were bothered by a problem, e.g. by having little interest or pleasure in doing things. Items are answered on a 4-point scale ranging from 0 = “not at all” to 3 = “nearly every day”.

Anxiety is assessed with the *Generalized Anxiety Disorder Questionnaire* (GAD-7; Williams, 2014). It assesses if the patient was bothered by complaints related to anxiety during the past 2 weeks, e.g. by asking about having trouble to relax. Items are answered on a 4-point Likert scale ranging from 0 = “not at all” to 3 = “nearly every day”.

The 11-item *Resilience Scale* (RS-11; Kocalevent et al., 2015) asks about general resilience towards life events, e.g. “I feel that I can handle many things at a time.”. Items are answered on a 7-point scale ranging from 1 = “strongly agree” to 7 = “strongly disagree”.

Insomnia, e.g. difficulty falling asleep, is assessed by the *Insomnia Severity Index* (ISI; Morin, 1993). Items are answered on a 5-point scale with regards to severity during the past 2 weeks, with wording differing between questions (e.g., from 0 = “very dissatisfied” to 4 = “very satisfied”).

Fatigue is assessed by the 11-item *Chalder Fatigue Scale* (Jackson, 2014), e.g. by asking “Do you have difficulty concentrating?”. Items are answered on a 4-point scale ranging from 0 = “Better than usual” to 3 = “much worse than usual”.

The *Perceived Stress Scale* (PSS; Cohen et al., 1983) investigates the experience of psychological stress in the past 4 weeks. It consists of 10 items, e.g., “In the last month, how often have you been upset because of something that happened unexpectedly?”. Items are answered on a 5-point scale ranging from 1 = “never” to 5 = “very often”.

EMA Questions: After each measurement point (BL, T1, T2, T3), patients will be asked to answer 8 daily EMA questions for 10 consecutive days. Questions will be completed in Redcap® with the survey link being sent at 6pm via SMS and Email. On a 10-point scale ranging from 0 = “not at all” to 9 = “severely”, Patients will answer the following questions: “Over the last 24h, how much have you been:

- bothered by feeling sad, down, or uninterested in life?
- bothered by feeling anxious or nervous?
- bothered by feeling stressed?
- bothered by feeling angry?
- bothered by not having the social support you feel you need?
- bothered by difficulties to pursue your daily activities?
- fully in the moment, accepting it as it is?
- high in self-compassion towards yourself?

The *System Usability Scale* (SUS; Brooke, 1996) will be administered at T1 after completing the DiLCoS intervention. The SUS assesses how easy it is to use the system, in this case the application. It consists of 10 items, e.g. “I think I would like to use the system often”. Items are answered on a 5-point scale ranging from 1 = “strongly disagree” to 5 = “strongly agree”.

Measures of physical performance:

To measure physical performance, the JAMAR® grip strength test, the 6-minute walking test, and the one minute sit to stand test will be conducted.

Grip strength will be assessed using the JAMAR®, a hydraulic grip strength measuring device (American Society for Surgery of the Hand, 1983). While sitting on a chair, the patient is instructed to squeeze the JAMAR® with one hand, while the other arm is resting in their lap. The test is repeated thrice for each hand, first for the dominant hand, then for the non-dominant hand. The best value (in kg) for each hand is taken. Further, injuries to one hand or both are noted.

The aim of the 6-minute walking test (Butland et al., 1982) is to assess functional exercise capacity by measuring the distance (in meters total) that a patient can walk in 6 minutes. Pulse and oxygen saturation is assessed at baseline, directly after finishing the test, and 2 minutes after finishing the test. Well-being before and after the test is assessed on a 5-point Likert-scale ranging from 1 = "very bad" to 5 = "very good". After the test, perceived exertion of breathing and legs is assessed with the Borg Category-Ratio (CR) 10 Scale ranging from 0 = "no exertion" to 10 = "maximal exertion" (Borg, 1998).

The one minute sit-to-stand test (STS-60; Koufaki et al., 2002) is a measure for muscular endurance. It assesses the number of sit-to-stand cycles that can be achieved within one minute. The patient is seated on a chair without armrests and to stand up and sit continuously for one minute, without using their hands as support. The number of sit-to-stand cycles as well as oxygen saturation after completing the test is assessed.

DiLCoS Substudy

The DiLCoS substudy usually starts after participants have completed the baseline (T0) measurements of the BALCoS study and before the T1 measurements. This timing ensures that the impact of the digital intervention can be isolated and accurately assessed within the broader scope of BALCoS. Due to a decrease in PCC referrals, patients who are further progressed in BALCoS (beyond T1) will also be invited to take part in the intervention. To evaluate their participation in the intervention, they will again complete the measures described in section above (with the last measure completed within 3 months before the start of the intervention being the new BL). As part of the onboarding process, participants will receive a text message sent from the DocDok system (see 7.3. Overview of the DocDok system) inviting them to install the docdok smartphone application from the Google Play Store or the iOS App Store. Included in this text message is an initial password for logging into the docdok smartphone application. Only with this password which is generated after patients are added to the study, patients can access the content of the application. After initial login, participants choose their own password and login with their personalized password from that moment. The smartphone application is equipped with a standard password renewal function, that allows participants to change their password if they have forgotten it. The study personnel facilitating the onboarding process will explain the structure of the digital intervention by going through a demonstration module together with the participant and answering questions/addressing concerns raised by the participant. The demonstration module contains a summary of the type of content that patients will receive during the intervention and a description of the structure of each module. Additionally, the module contains the remark that the primary aim of the smartphone application is clinical research. Paitents are also reminded that they should contact the study personnel whenever they feel uncomfortable with an exercise and that they can skip or stop exercises at any point of the intervention. After completion of said demonstration, the participant and the study team will decide an appropriate starting date, with possible start days to be either Thursday or Friday.

DiLCoS is a 12-week program with an estimated daily time commitment of approximately 15 minutes. The intervention material is organized in 11 modules with a duration of seven days each. All 11 modules start with an introduction and a short meditation as a starting ritual. Two of these modules are wrap-up weeks (module 4 and 8), which means that patients repeat the content of previous modules. Module 9 will be one out of three different modules chosen at study enrolment: Depending on individual symptoms, the study personnel will decide together with the patients which of these modules will be allocated to them. This ensures a personalized support. At the start of each new module, participants receive a notification. They additionally receive one daily reminders via the smartphone application to engage with the digital intervention. On days 1 – 4 of each module, participants are usually provided with psychoeducational elements (e.g., information about stress), exercises (e.g. breathing exercises), and guided reflections (e.g.,

construction of an action plan with feedback on the application). Some modules will include psychometric questionnaires, however, the score will not be computed automatically. Instead, participants will be provided material and instruction in order to do so by themselves. On days 5 and 7 of each module, usually no additional or new content is provided. Those days are rest days or provide the opportunity to repeat previous content from the current module. Encouraging the participants to repeat parts of the material enables them to gain proficiency and foster their progress in areas where they can benefit most. On day 6, standardized feedback questions are asked about the module (see case report form; CRF). These feedback questions provide insight into user perceptions of the intervention's feasibility, comprehensiveness, effectiveness, and scope (e.g., length and depth of the material). After the questions, a conclusionary statement follows. The content of module 4 and 8 is solely based on the individual participant's feedback to questions 3 and 4. This feedback mechanism furthermore promotes reflection and encourages active engagement on the side of the participant. At the beginning of the digital intervention, a four-day introduction module (with day 4 being a rest day) will be provided. Likewise, a conclusion module will be provided at the very end of the intervention. See Figure 2 for an overview on the intervention structure and module content and Appendix 3 for screenshots illustrating the layout of the smartphone application and the platform where patients are added to the study by the study personnel.

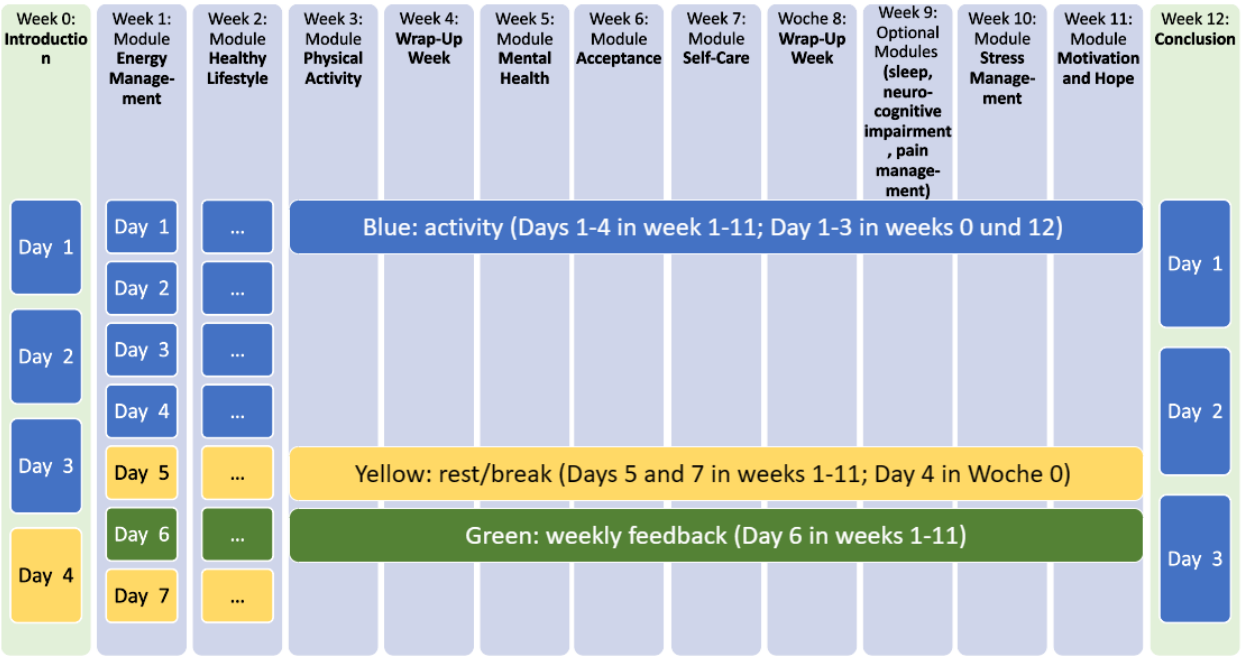

Figure 2: Overview of modules and their structure.

Detailed information on the module content

Module 0: Introduction

The aim of this module is to act as a frame for the intervention. In this module, participants are introduced to the intervention content, structure and its goals. Additionally, the participants are informed about how to contact the study team and are provided with resources in case of emergencies (a collection of phone numbers for emergency services).

Module 1: Energy Management

This module focuses on fatigue as the most common symptom of PCC and how post-exertional malaise (PEM) can impact daily life. Participants are presented with exercises on how to monitor and better manage their energy levels, and how to deal more effectively with fatigue or exhaustion. Patients are instructed to only do exercises that their energy levels allow and, if in doubt, contact the study personnel or skip the exercise.

Module 2: Healthy Lifestyle

This module focuses on healthy nutrition in general and in the context of PCC, as well as mindful media consumption and exposure to information about PCC and their possible impacts.

Module 3: Physical Activity

This module focuses on how physical activity can be adjusted in order to better manage PCC symptoms. It covers strategies (i.e. pacing) and includes a video series about breathing techniques and relief positions for easier breathing, as well as exercises to manage physical symptoms and adjust activities of daily living in order to better deal with PCC related impairments.

Module 4: Wrap-Up Week 1

The content for this module is based on the feedback of the participants about the exercises from modules 1, 2 and 3 (see Standardized feedback questions; CRF). Those exercises that have been evaluated as most useful to the individual participant are sent via chat function for them to repeat. Participants also have the opportunity to complete unfinished modules during this time.

Module 5: Mental Health

This module consists of a video series about steps to improve mental health and covers the topics of anxiety, depression, management of physical symptoms, change of unwelcome thoughts and the power of social support in the context of PCC. It focuses on supporting participants to build better resilience and add to their strategies and techniques in dealing with PCC related psychological challenges.

Module 6: Acceptance

Based on principles of Acceptance- and Commitment Therapy (ACT), four key elements of acceptance are presented (acceptance and avoidance, understanding of cognitive fusion, the inner observer, and the value compass). The module offers exercises and promotes self-reflection regarding the participants' situation and condition.

Module 7: Selfcare

This module focuses on the importance of self-care and self-compassion and how to overcome the “inner critic”. It contains a guided reflection exercises about their individual impairment caused by PCC and how they can better deal with the negative thoughts and emotions connected to it. A written exercise furthermore helps them distance themselves from the negative impact of PCC and presents them with a technique to better practice selfcare in their daily lives and activities.

Module 8: Wrap-Up Week 2

Similarly to Module 4, this module's content is based on participants' feedback on the exercises of Modules 5, 6, and 7. The exercises evaluated as most helpful and/or useful are sent via the chat function.

Module 9a: Sleep

Participants are presented with the core principles of sleep hygiene. Exercises to deal with sleep disorders (e.g. insomnia, parasomnia) and relaxation techniques to more easily fall sleep are also presented. Participants are encouraged to try out these exercises if necessary and applicable to their situation.

Module 9b: Neurocognitive Impairment

This module focuses on exercises to train and improve brain functions that are commonly impaired by PCC (i.e. memory, working memory, concentration and attention, and executive functions). Participants are guided through the variations and options regarding a wide variety of possible exercises in terms of choice, planning, execution and reflection.

Module 9c: Pain Management

Participants allocated to this module are presented with information relating to the mechanisms of pain and how it relates to PCC. The module contains additional information regarding strategies of pain management and relaxation exercises and participants are reminded of such exercises that they have encountered in past modules (e.g. breathing exercises and relief positions in

Module 3). They are guided through choosing, planning, executing and providing feedback about this content.

Module 10: Stress Management

This module focuses on different types of stress and the consequences of it. Participants are guided through evidence-based techniques in stress management (i.e. progressive muscle relaxation (PMR) and are presented with two exercises designed to recognize stressors and adjust their behavior accordingly to minimize stressful situations and improve overall well-being.

Module 11: Motivation and Hope

This module focuses on expectations regarding symptoms and the future course of PCC. Participants are instructed to imagine their lives in a year and are guided through steps to better adjust their prediction and manage their expectations in relation to PCC with the goal of having a more positive view on the future.

Module 12: Conclusion

This module concludes the digital intervention. Participants are asked to review the past weeks and months and reflect on their progress, the changes and the different techniques, strategies and psychoeducational content they have encountered. Furthermore, they are presented with a 10-step guide that is designed to help make meaningful and lasting changes to their habits and to better adjust to PCC.

3.4 Withdrawal and discontinuation

Participants' participation in the study is voluntary and participants can withdraw their consent at any time without giving reasons. Their treatment/routine care will not be affected by this decision. Patients who drop out before T3 will receive a proportionate compensation of the CHF 50 they receive after completing T3. Patients completing all measures a second time (see 3.1) will receive this compensation again. Patients who withdraw their study consent/who drop out of the study will be asked for consent to analyze their encoded data up to the point of consent withdrawal/dropping out of the study. If they decline, their data will be deleted. After analyses, personal information of withdrawn participants (stored in a separate, password-protected document) will be deleted.

DiLCoS Substudy

Participation in the substudy is voluntary and consent can be withdrawn at any time without providing reasons. Patients' treatment/routine care will not be affected by this decision and it will not affect their participation in the larger BALCoS study itself. Patients who withdraw substudy consent or who drop out of the substudy will be offboarded from the platform and can no longer access study material or log in to the smartphone application. Patients who withdraw their study consent/who drop out of the study will be asked for consent to analyze their encoded data up to the point of consent withdrawal/dropping out of the study. This also means that their registration data will stay on the platform until the final data export, however, they will not be contacted again. If they decline, all their data will be deleted.

4 STATISTICS AND METHODOLOGY

4.1. Statistical analysis plan

As this is a registry-based cohort study, no formal sample size calculation is required. In the PCC outpatient clinic at the UHB, a total of 173 patients has been seen during the first year. Due to the constant changes in SARS-CoV-2 viral variants and the changing immune status of the population, it is not possible to definitely predict how many patients will visit the PCC outpatient clinic in the future. At this time, similar numbers of patients per year and consent of approximately 80% of patients are anticipated, resulting in around N=136 patients per year enrolled in BALCoS.

Data will be analyzed in RStudio (Racine, 2012) or other statistical software packages. For baseline comparisons, continuous data will be reported as mean +/- standard deviation or median (interquartile range) and assessed means of analyses of variance (ANOVA), using general linear model (GLM)-family based procedures and Mann-Whitney U tests, depending on the variable distribution. Categorical variables will be described as counts (percentage) and compared using chi-square tests. For longitudinal and other data analyses, state-of-the-art procedures, such as generalized (mixed) models will be calculated to assess both within- as well as between-patient changes.

DiLCoS Substudy

A power analysis was performed using G*Power 3.1 (Faul et al., 2007). Assuming a power (1-ß) of 0.80, α = 0.05, and a small to medium effect size of f = 0.15 in a repeated measures, within factors ANOVA with two time points resulted in N=105 participants. This estimation took a possible dropout rate of 15% into account. To have sufficient power for secondary analyses, this sample size is considered as the minimal sample size and more patients will be enrolled, if feasible during the data collection phase (first patient in March 2024, first patient out February 2025; last patient in March 2025, last patient out March 2026).

The primary analysis is a pre-post comparison of the primary and secondary endpoints (see section 2.2 Primary and secondary endpoints), with T0 as a pre-intervention measure and T1 as a post-intervention measure.

As a secondary analyses, T2 and T3 will be analyzed as follow-up measures to investigate the time-stability of intervention effects. Further, a comparative analysis (DiLCoS - treatment as usual) may be performed: Patients who participated in the BALCoS study before the initiation of the DiLCoS substudy as well as patients who agree to participate in BALCoS but do not give consent to participate in DiLCoS will be considered as a non-randomised control group. This will help in distinguishing the specific effects of the DiLCoS intervention to get a comprehensive understanding of the efficacy of the digital intervention. With these two groups, generalized mixed models will be calculated to assess both within- as well as between-patient changes.

Lastly, results from the DiLCoS substudy might serve as an additional arm to another intervention study conducted within the EU consortium (AIR-program and HUS Internet Therapy Compared to Treatment as Usual in Functional Disorders and Post Covid-19 Condition; https://www.clinicaltrials.gov/study/NCT05212467), using a benchmarking controlled trial approach (Malmivaara, 2015). To do so, encoded data will be transferred to the "acamedic" platform (see section 7.4 Confidentiality and coding).

4.2. Handling of missing data

Missing data will be handled by appropriate techniques, such as multiple imputation or mixed effect models. To reduce missings, the scientific importance of conscientious participation and complete filling in of questionnaires will be explained to patients. Using digital assessment tools will further reduce missing data. Study personnel will monitor the completion of measures and will identify problems as early as possible.

DiLCoS Substudy

Participation in the intervention will be monitored by study personnel. If participants do not visit the smartphone application for more than two days, they will be contacted via the applications' chat function. If there is no activity for an additional two-day period, they will be contacted via phone to solve potential problems. Participants who attended less than 50% of the intervention weeks will be excluded from completer analyses.

5 REGULATORY ASPECTS AND SAFETY

5.1 Local regulations / Declaration of Helsinki

This research project will be conducted in accordance with this study protocol, the Declaration of Helsinki (World Medical Association, 2013), the principles of Good Clinical Practice, the Human Research Act (HRA; The Swiss Federal Council), and the Human Research Ordinance (HRO; Federal Office of Public Health, 2022) as well as other locally relevant regulations. Both the project leader and the sponsor acknowledge their responsibilities.

5.2 Notification of safety and protective measures (HRA Art. 15, HRO Art. 20)

If, during the research project, circumstances arise which could jeopardize the safety or health of the participants or lead to a disproportionate relationship between the risks or burdens and the benefits, all the measures required to ensure protection are to be taken without delay.
The project leader and the sponsor are promptly notified (within 24 hours) if immediate safety and protective measures have to be taken during the conduct of the research project. The Ethics Committee will be notified via BASEC of these measures and of the circumstances necessitating them within 7 days.

5.3 Serious events (HRO Art. 21)

If a serious event occurs, the research project will be interrupted and the Ethics Committee notified of the circumstances via BASEC within 7 days according to HRO Art. 21[1].
Patients will be recruited from an outpatient setting; participating in the proposed study does not influence standard care. We do not expect any SAEs to occur as a consequence of the tests administered for this study in addition to standard care.

5.4 Procedure for investigations involving radiation sources

Not applicable.

5.5 Amendments

Substantial changes to the project set-up, the protocol, and relevant project documents will be submitted to the Ethics Committee for approval according to HRO Art. 18 before implementation. Exceptions are measures that have to be taken immediately in order to protect the participants.

5.6 End of project

Upon project completion or discontinuation, the Ethics Committee will be notified within 90 days. All biological materials and health-related data are encoded upon termination of data analysis (also refer to section 7.4). If the respective consent is given (see consent form), the biological material (blood) will be stored in the biobank at the UHB and can be used in follow-up/additional projects. All other samples will be destroyed.

5.7 Insurance

In the event of project-related damage or injuries, the Sponsor will be liable (covered by the liability insurance of USB), except for damages that are only slight and temporary; and for which the

[1] A serious event is defined as any adverse event where it cannot be excluded, that the event is attributable to the sampling of biological material or the collection of health-related personal data, and which:
a. requires inpatient treatment not envisaged in the protocol or extends a current hospital stay;
b. results in permanent or significant incapacity or disability; or
c. is life-threatening or results in death.

extent of the damage is no greater than would be expected in the current state of scientific knowledge (Art. 12 HRO).

6 FURTHER ASPECTS

6.1 Overall ethical considerations

As this study is designed as a registry-based cohort study with a digital intervention substudy, there is little to none potential unethical burden on the participants. This study's conduction will comply with the Declaration of Helsinki (World Medical Association, 2013), the ICH-GCP, and the HRA. Additionally, the EU consortium has dedicated a work package to ethical and privacy considerations and has hired a company to advise and supervise issues regarding data privacy, secure data sharing, ethics of the project, and regulatory compliance within the project. For further justification of the project, refer also to section 1, section 6.2, and the informed consent form.

For ethical considerations regarding the intervention content, refer to section 6.2.

6.2 Risk-Benefit Assessment

The study has strong potential benefits for future PCC patients, hospital staff, and stakeholders by contributing to the knowledge of the pathophysiology of PCC and to a better understanding of risk factors and predictors of PCC. This might also inform treatment options for PCC. A part of the patients included in the cohort study are given the opportunity to participate in the DiLCoS substudy. Participation in the study is not associated with any risks. Completing the neurocognitive testing and the measures of physical performance might be mildly tiring. Having more blood drawn than needed for routine assessment might cause mild discomfort. By participating in the questionnaires or conversations, problem areas can be discussed for the benefit of the patient, which however can also be perceived as unpleasant for a short time. We do not expect any negative effects or damage from routine care. All applied questionnaires are validated, widely accepted, and routinely applied in research and clinical practice. Thus, it can be concluded that the risk-benefit assessment is positive.

DiLCoS Substudy

Safety Considerations

To communicate with the study personell, participants are instructed to use the chat function on the smartphone application. Should the chat function not be available to them due to technical problems, they are informed that they can contact the study team via email. Before any mental or physical exercise, participants are informed that they can stop or interrupt at any time during an exercise should they feel unwell, uncomfortable with the material, experience adverse reactions, or feel negatively impacted in any way by the material provided. They are also advised to seek medical counsel if they feel unable or unsure to execute physical exercises and execute said physical exercise under medical supervision. Participants are instructed that the chat function/study email is not meant for emergency situations. For the unlikely case that an emergency situation arises, participants receive resources in the introduction module with emergency phone numbers, should they feel overwhelmed or negatively impacted by the study material. This is implemented as a safety measure and to ensure that participants engage only in those exercises that are within their capabilities at the time.

Risks

- Additional exposure to information about PCC might increase cognitive load and worrysome thoughts or dispositions.
- Exercises (for example breathing exercises) will be shown how to do correctly, but participants will execute exercises without supervision. Patients are encouraged to execute said exercises in a safe and health-conscious manner and are informed that the exercises might be interrupted at any time should the need arise.

Benefits

- Patients receive a 12-week programm based on evidence-based treatment recommendations that consider expertise from different specialties (psychosomatics, internal medicine, physical therapy) with access to individual care via chat function on the smartphone application.
- Patients can acquire specific skills and ressources to better deal with burdening symptoms and improve their health and well-being.
- Patients can gain a better understanding of disease course and symptoms.

To conclude, given the need for effective interventions for PCC, the risk-benefit assessment is positive.

6.3 Rationale for the inclusion of vulnerable participants

Pregnancy does not affect any study procedures. There will not be any specific risk in relation to pregnancy.

7 QUALITY CONTROL AND DATA PROTECTION

7.1 Quality measures

Data quality is assured through (1) the investigators who are qualified by education, experience, and extensive training (data collection, data entry); (2) continuous monitoring, quality control, and plausibility check of collected data by data managers; (3) internal audit procedures that will guarantee the quality of the findings. Data management will be performed only by authorized and experienced study investigators and project employees.

For quality assurance, the Ethics Committee may visit the research sites. Direct access to the source data and all project-related files and documents will be granted on such occasions.

7.2 Data recording and source data

Data will be collected mainly online via Redcap®, some by paper-pencil (informed consent form, physiotherapeutic assessment) and within the CNSVS program. All collected data and hospital information that is not electronically available will be entered into the study electronic case report form (eCRF) of Redcap®. Data entry will be performed by trained study personnel. Password protection and user-right management ensure that only authorized study investigators, monitors, data managers, and local authorities (if necessary) will have access to the data during and after the study. Back-up of the Redcap® database server will be performed regularly according to established processes by the ICT-department of the UHB. When data gets saved in an eCRF, it will be validated for completeness and discrepancies by a data manager. Clinical data will be retrieved from the patient records and merged in the eCRF with data from Redcap®.

DiLCoS Substudy

Data will be collected via the smartphone application and is only accessible through a secure login on the DocDok digital platform. All collected data will be encoded before any additional analysis. Logins are individualized and password-protected to ensure data safety and traceability of access on the webpage. Data collected from the smartphone application will be hosted in Switzerland (server location and registered seat of partner company Docdok), with some employees of the company working from Israel (safe third country according to EU adequacy decision; registered seat of subsidiary of partner company).

To understand how users interact with the smartphone application, the following data will be collected:

- Frequency of Use: Measuring how often users engage with the application.
- Feature Utilization: Identifying which parts of the application are most frequently used.

- User Engagement Duration: Tracking the amount of time spent on the application during each session.
- Navigation Patterns: Observing how users move through different sections of the application.

This data will be collected using the following methods:

- Automated Tracking Tools: Embedded in the software of the application to track user interactions.
- User Feedback: Questions presented to users for additional qualitative feedback (see Section 3.3 Study Procedures and CRF)

7.3 Overview of the DocDok System

To provide the intervention within the DiLCoS substudy, we utilize a delivery platform provided by DocDok.Health. DocDok is a Swiss software company that provides efficient and secure management of digital health content, ensuring a seamless user experience, which has been successfully used in different Swiss healthcare facilities (University Hospital of Zurich (USZ), Lausanne University Hospital (CHUV, Spital STS AG). Their server is located in Switzerland. Data can be downloaded from their server and will then be added to the eCRF of Redcap® by the study personnel.

The DocDok system is a state-of-the-art digital platform that has been specifically tailored for the healthcare sector. It offers a comprehensive suite of features designed to enhance the delivery and management of health-related content on smartphone devices. Key aspects of the DocDok system include:

- System structure: The docdok system is comprised of a smartphone application and web-based access. Users can access the system by login into the application and health care professionals access the system by login into the web-based platform, both with secure logins. This makes the docdok system easily accessible for both user groups.
- Security and Compliance: The system adheres to stringent security protocols, ensuring the confidentiality and integrity of user data. It is compliant with the Swiss Data Proception Law and GDPR standards in Europe, providing a secure framework for handling sensitive health information. Hosting of data is provided in Switzerland.
- User Interface and Experience: DocDok boasts an intuitive and user-friendly interface, facilitating easy navigation and accessibility for users of varying technological proficiency. This enhances user engagement and ensures effective content delivery.
- Customization Capabilities: The system offers extensive customization options, allowing for the tailoring of content and features to meet the specific needs of different user groups, including patients with PCC.
- Real-time Responses: The platform allows responding to user engagement and information provided by the user.
- Support and Maintenance: Ongoing technical support and regular updates are provided to ensure the system remains current with the latest technological advancements and security updates, ensuring uninterrupted service and reliability.
- Engagement and Interaction Dashboard: This dashboard is specifically designed to track and facilitate interactions with patients enrolled in the study. It serves as a dynamic tool for monitoring patient engagement and enabling direct communication. The dashboard provides real-time data on patient interaction with specific content. It thereby acts as interactive communication platform, serving as a two-way communication channel between study personnel and participants. Through this platform, participants can receive personalized messages, reminders, and feedback, while also having the ability to send feedback or queries.

7.4 Confidentiality and coding

Project data will be handled with the uttermost discretion and is only accessible to authorized personnel who require the data to fulfil their duties within the scope of the research project. On the CRFs and other project specific documents, participants are only identified by a unique

participant number. Only to send out the questionnaires at T1 and T2 and for the EMA assessment, patients' email address and phone number will be recorded in Redcap®. The email address and phone number will not be included in the data set exported for statistical analyses.
The list of participants with given consent will be stored in a secure UHB server maintained by the ICT-department and the trial and participant data will be handled with uttermost discretion and is only accessible to authorized personnel, who needs data access to fulfill their duties within the scope of the study. Back-up of the UHB server will be performed regularly according to established processes by the ICT-department of the UHB. As soon as possible after data collection, the CRFs and other study-specific documents are encoded and the participants are henceforth only identified by a unique participant number. The complete dataset will be exported and transferred to those analysing the data as well as the principal investigator through a secure channel. Other members of the study team will receive access to the data, as required for analytical tasks. For data preservation, eCRF data will be exported and stored as comma-separated value (*.csv) files. Data will further be transferred to the "Acamedic" platform at HUS Helsinki University Hospital (described separately in the Joint Controllership Agreement).

Biological material in this project is not identified by participant name but by a unique participant number. Biological material is appropriately stored in a restricted area on hospital premises only accessible to authorized personnel.
Biological materials for consortium partners collected during the research project will be shipped outside the research site (Finland, Germany, and Estonia). Specimen will be shipped on dry ice by a service specialized in medical delivery. Please also refer to the respective SOP protocols attached to this protocol as well as to the informed consent form. For data collected during the research project that are to be transferred outside the research site, please refer to the Joint Controllership Agreement attached to this protocol.

DiLCoS Substudy
For enrollment in the substudy, participants' email address and phone number are required in order for them to receive login information for the smartphone application. Furthermore, the following information is required to add participants to the docdok system: saluation or title, first name, last name, sex, and date of birth. Their information will be accessible by themselves and the authorized members of the study team, since their information will be located in the docdok system until they finish with the intervention. Data collected will be encoded before analysis or where appropriate recoded to their unique identifier within BALCoS.

7.5 Retention and destruction of project data and biological material

Health-related data are stored for 10 years after publication of the research project. Biological materials (blood samples) sent to consortium partners will be used entirely for the planned analyses and thus not stored afterwards.
At the UHB, samples will be stored for at least 10 years. If appropriate consent is granted, the samples not used in BALCoS will be bio-bagged and may be used in future studies.
Data from DiLCoS will be archived for 20 years.

8 FUNDING / PUBLICATION / DECLARATION OF INTEREST

BALCoS is primarily funded by the Swiss State Secretariat for Education, Research and Innovation (SERI) under contract number 22.00094 and by the European Union's Horizon Europe research and innovation programme under grant agreement No 101057553. The project leaders at UHB declare that they have no conflict of interest regarding the project funding. The funding source had no impact on the design of this study and will not influence its execution, analyses, interpretation of the data, or decision to submit publications. The research activities are fully independent and there are no intellectual or financial proprietary claims. Data privacy and sharing policies are supported by EU-consortium partners (NUROMEDIA GMBH and Chino.io), who ensure a secure transfer of data to the "Acamedic" platform (Helsinki, Finland) and General Data

Protection Regulation (GDPR) compliance of data sharing by setting up a data sharing agreement (Joint Controllership Agreement; submitted in a separate document). The cooperation between University Hospital Basel and the provider of the software used to provide the DiLCoS application (DocDok.health) is based on an respective offer (see separate documents).

9 REFERENCES

American Society for Surgery of the Hand. (1983). *The Hand: Examination and Diagnosis* (2nd ed.). Churchill Livingstone, Edinburgh.

Ballering, A. V., van Zon, S. K. R., olde Hartman, T. C., & Rosmalen, J. G. M. (2022). Persistence of somatic symptoms after COVID-19 in the Netherlands: an observational cohort study. *The Lancet, 400*(10350), 452-461. https://doi.org/10.1016/S0140-6736(22)01214-4

Blanchard, M., Backhaus, L., Ming Azevedo, P., & Hügle, T. (2022, Feb 4). An mHealth App for Fibromyalgia-like Post-COVID-19 Syndrome: Protocol for the Analysis of User Experience and Clinical Data. *JMIR Res Protoc, 11*(2), e32193. https://doi.org/10.2196/32193

Borg, G. (1998). *Borg's perceived exertion and pain scales*. Human Kinetics.

Brooke, J. (1996). SUS-A quick and dirty usability scale. *Usability evaluation in industry, 189*(194), 4-7.

Butland, R. J., Pang, J., Gross, E. R., Woodcock, A. A., & Geddes, D. M. (1982, May 29). Two-, six-, and 12-minute walking tests in respiratory disease. *Br Med J (Clin Res Ed), 284*(6329), 1607-1608. https://doi.org/10.1136/bmj.284.6329.1607

Cohen, S., Kamarck, T., & Mermelstein, R. (1983, Dec). A global measure of perceived stress. *J Health Soc Behav, 24*(4), 385-396.

Crook, H., Raza, S., Nowell, J., Young, M., & Edison, P. (2021). Long covid—mechanisms, risk factors, and management. *BMJ, 374*, n1648. https://doi.org/10.1136/bmj.n1648

Cuijpers, P., Heim, E., Ramia, J. A., Burchert, S., Carswell, K., Cornelisz, I., Knaevelsrud, C., Noun, P., van Klaveren, C., Van't Hof, E., Zoghbi, E., van Ommeren, M., & El Chammay, R. (2022, Dec). Guided digital health intervention for depression in Lebanon: randomised trial. *Evid Based Ment Health, 25*(e1), e34-e40. https://doi.org/10.1136/ebmental-2021-300416

Dalbosco-Salas, M., Torres-Castro, R., Rojas Leyton, A., Morales Zapata, F., Henríquez Salazar, E., Espinoza Bastías, G., Beltrán Díaz, M. E., Tapia Allers, K., Mornhinweg Fonseca, D., & Vilaró, J. (2021). Effectiveness of a Primary Care Telerehabilitation Program for Post-COVID-19 Patients: A Feasibility Study. *Journal of Clinical Medicine, 10*(19), 4428. https://www.mdpi.com/2077-0383/10/19/4428

Dang, A., Dang, D., & Rane, P. (2021). The Expanding Role of Digital Therapeutics in the Post-COVID-19 Era.

Digital Therapeutics Alliance. (2019). *Digital Therapeutics: Definition and Core Principles* https://dtxalliance.org/wp-content/uploads/2021/01/DTA_DTx-Definition-and-Core-Principles.pdf

El-Toukhy, S., Hegeman, P., Zuckerman, G., Anirban, R. D., Moses, N., Troendle, J. F., & Powell-Wiley, T. M. (2023, Dec 7). A prospective natural history study of post acute sequalae of COVID-19 using digital wearables: Study protocol. *Res Sq*. https://doi.org/10.21203/rs.3.rs-3694818/v1

Faul, F., Erdfelder, E., Lang, A.-G., & Buchner, A. (2007). G*Power 3: A flexible statistical power analysis program for the social, behavioral, and biomedical sciences. *Behavior Research Methods, 39*(2), 175-191. https://doi.org/10.3758/BF03193146

Federal Office of Public Health. (2022). *COVID-19 Switzerland: Information on the current situation, as of 18 October 2022* https://www.covid19.admin.ch/en/overview?time=total

Gualtieri, C. T., & Johnson, L. G. (2006). Reliability and validity of a computerized neurocognitive test battery, CNS Vital Signs. *Archives of Clinical Neuropsychology, 21*(7), 623-643. https://doi.org/10.1016/j.acn.2006.05.007

Harenwall, S., Heywood-Everett, S., Henderson, R., Godsell, S., Jordan, S., Moore, A., Philpot, U., Shepherd, K., Smith, J., & Bland, A. R. (2021, Jan-Dec). Post-Covid-19 Syndrome: Improvements in Health-Related Quality of Life Following Psychology-Led Interdisciplinary Virtual Rehabilitation. *J Prim Care Community Health, 12*, 21501319211067674. https://doi.org/10.1177/21501319211067674

Jackson, C. (2014). The Chalder Fatigue Scale (CFQ 11). *Occupational Medicine, 65*(1), 86-86. https://doi.org/10.1093/occmed/kqu168

Kocalevent, R.-D., Zenger, M., Heinen, I., Dwinger, S., Decker, O., & Brähler, E. (2015). Resilience in the General Population: Standardization of the Resilience Scale (RS-11). *PLOS ONE, 10*(11), e0140322. https://doi.org/10.1371/journal.pone.0140322

Kortianou, E. A., Tsimouris, D., Mavronasou, A., Lekkas, S., Kazatzis, N., Apostolara, Z. E., Isakoglou, M., Dimakou, G., Barmparessou, Z., Tsikrika, S., Sakka, V., Liontos, A., Christaki, M., Milionis, H., & Kalomenidis, I. (2022). Application of a home-based exercise program combined with tele-rehabilitation in previously hospitalized patients with COVID-19: A feasibility, single-cohort interventional study [journal article]. *Pneumon, 35*(2), 1-10. https://doi.org/10.18332/pne/146521

Koufaki, P., Mercer, T. H., & Naish, P. F. (2002). Effects of exercise training on aerobic and functional capacity of end-stage renal disease patients. *Clinical Physiology and Functional Imaging, 22*(2), 115-124. https://doi.org/https://doi.org/10.1046/j.1365-2281.2002.00405.x

Kroenke, K., Spitzer, R. L., & Williams, J. B. W. (2002). The PHQ-15: Validity of a New Measure for Evaluating the Severity of Somatic Symptoms. *Psychosomatic Medicine, 64*(2), 258-266. https://journals.lww.com/psychosomaticmedicine/Fulltext/2002/03000/The_PHQ_15__Validity_of_a_New_Measure_for.8.aspx

Kroenke, K., Strine, T. W., Spitzer, R. L., Williams, J. B. W., Berry, J. T., & Mokdad, A. H. (2009, 2009/04/01/). The PHQ-8 as a measure of current depression in the general population. *Journal of Affective Disorders, 114*(1), 163-173. https://doi.org/https://doi.org/10.1016/j.jad.2008.06.026

Krotz, A., Sosnowsky-Waschek, N., Bechtel, S., Neumann, C., Lohkamp, M., Kovacs, G., Genser, B., & Fischer, J. E. (2023, 2023/12/08). Reducing sick leave, improving work ability, and quality of life in patients with mild to moderate Long COVID through psychosocial, physiotherapeutic, and nutritive supportive digital intervention (MiLoCoDaS): study protocol for a randomized controlled trial. *Trials, 24*(1), 798. https://doi.org/10.1186/s13063-023-07819-7

Kumar, N., Khunger, M., Gupta, A., & Garg, N. (2015, Feb). A content analysis of smartphone-based applications for hypertension management. *J Am Soc Hypertens, 9*(2), 130-136. https://doi.org/10.1016/j.jash.2014.12.001

Li, J., Xia, W., Zhan, C., Liu, S., Yin, Z., Wang, J., Chong, Y., Zheng, C., Fang, X., Cheng, W., & Reinhardt, J. D. (2022, Jul). A telerehabilitation programme in post-discharge COVID-19 patients (TERECO): a randomised controlled trial. *Thorax, 77*(7), 697-706. https://doi.org/10.1136/thoraxjnl-2021-217382

Loft, M. I., Foged, E. M., & Koreska, M. (2022). An Unexpected Journey: The Lived Experiences of Patients with Long-Term Cognitive Sequelae After Recovering from COVID-19. *Qualitative Health Research, 32*(8-9), 1356-1369. https://doi.org/10.1177/10497323221099467

Lopez-Leon, S., Wegman-Ostrosky, T., Perelman, C., Sepulveda, R., Rebolledo, P. A., Cuapio, A., & Villapol, S. (2021, 2021/08/09). More than 50 long-term effects of COVID-19: a systematic review and meta-analysis. *Scientific Reports, 11*(1), 16144. https://doi.org/10.1038/s41598-021-95565-8

Malmivaara, A. (2015, 2015/05/19). Benchmarking Controlled Trial—a novel concept covering all observational effectiveness studies. *Annals of Medicine, 47*(4), 332-340. https://doi.org/10.3109/07853890.2015.1027255

Menges, D., Ballouz, T., Anagnostopoulos, A., Aschmann, H. E., Domenghino, A., Fehr, J. S., & Puhan, M. A. (2021). Burden of post-COVID-19 syndrome and implications for healthcare service planning: A population-based cohort study. *PLOS ONE, 16*(7), e0254523. https://doi.org/10.1371/journal.pone.0254523

Merad, M., Blish, C. A., Sallusto, F., & Iwasaki, A. (2022). The immunology and immunopathology of COVID-19. *Science, 375*(6585), 1122-1127. https://doi.org/doi:10.1126/science.abm8108

Morin, C. M. (1993). Insomnia severity index.

Mueller, M. R., Ganesh, R., Hurt, R. T., & Beckman, T. J. (2023, Jul). Post-COVID Conditions. *Mayo Clin Proc, 98*(7), 1071-1078. https://doi.org/10.1016/j.mayocp.2023.04.007

Nalbandian, A., Sehgal, K., Gupta, A., Madhavan, M. V., McGroder, C., Stevens, J. S., Cook, J. R., Nordvig, A. S., Shalev, D., Sehrawat, T. S., Ahluwalia, N., Bikdeli, B., Dietz, D., Der-Nigoghossian, C., Liyanage-Don, N., Rosner, G. F., Bernstein, E. J., Mohan, S., Beckley, A. A., Seres, D. S., Choueiri, T. K., Uriel, N., Ausiello, J. C., Accili, D., Freedberg, D. E., Baldwin, M., Schwartz, A., Brodie, D., Garcia, C. K., Elkind, M. S. V., Connors, J. M., Bilezikian, J. P., Landry, D. W., & Wan, E. Y. (2021, 2021/04/01). Post-acute COVID-19 syndrome. *Nature Medicine, 27*(4), 601-615. https://doi.org/10.1038/s41591-021-01283-z

Nehme, M., Diem, L., Bassetti, C. L. A., & Guessous, I. (2023, 09/18). Swiss recommendations for the diagnosis, management and follow-up of post-COVID condition in primary care medicine (2023). *Swiss Medical Weekly, 153*(9), 3468. https://doi.org/10.57187/s.3468

NICE. (2022). *COVID-19 rapid guideline: managing the longterm effects of COVID-19* https://www.nice.org.uk/guidance/ng188/resources/covid19-rapid-guideline-managing-the-longterm-effects-of-covid19-pdf-51035515742

Nittas, V., Puhan, M., Gao, M., & West, E. (2021). Long COVID: Evolving Definitions, Burden of Disease and Socio-Economic Consequences. *Swiss School of Public Health*.

Norton, A., Olliaro, P., Sigfrid, L., Carson, G., Paparella, G., Hastie, C., Kaushic, C., Boily-Larouche, G., Suett, J. C., & O'Hara, M. (2021). Long COVID: tackling a multifaceted condition requires a multidisciplinary approach. *The Lancet Infectious Diseases, 21*(5), 601-602. https://doi.org/10.1016/S1473-3099(21)00043-8

O'Hare, A. M., Vig, E. K., Iwashyna, T. J., Fox, A., Taylor, J. S., Viglianti, E. M., Butler, C. R., Vranas, K. C., Helfand, M., Tuepker, A., Nugent, S. M., Winchell, K. A., Laundry, R. J., Bowling, C. B., Hynes, D. M., Maciejewski, M. L., Bohnert, A. S. B., Locke, E. R., Boyko, E. J., Ioannou, G. N., & Collaboratory, V. C. O. R. (2022). Complexity and Challenges of the Clinical Diagnosis and Management of Long COVID. *JAMA Network Open, 5*(11), e2240332-e2240332. https://doi.org/10.1001/jamanetworkopen.2022.40332

Peiris, S., Izcovich, A., Ordunez, P., Luciani, S., Martinez, C., Aldighieri, S., & Reveiz, L. (2023). Challenges to delivering evidence-based management for long COVID. *BMJ Evidence-Based Medicine, 28*(5), 295-298. https://doi.org/10.1136/bmjebm-2023-112311

Prashar, J. (2023). Long Covid: conceptualizing the challenges for public health. *Journal of Public Health, 45*(3), 771-779. https://doi.org/10.1093/pubmed/fdac153

Racine, J. S. (2012). RSTUDIO: A PLATFORM-INDEPENDENT IDE FOR R AND SWEAVE. *Journal of Applied Econometrics, 27*(1), 167-172. http://www.jstor.org/stable/41337225

Rafael Post-Covid Platform. (2023). *POST-COVID RECOMMENDATIONS FOR PRIMARY CARE PHYSICIANS* https://www.rafael-postcovid.ch/sites/default/files/inline-files/HUG-POSTCOVID-EN%20-A4-FINAL_PROD.pdf

Rinn, R., Gao, L., Schoeneich, S., Dahmen, A., Anand Kumar, V., Becker, P., & Lippke, S. (2023, Apr 17). Digital Interventions for Treating Post-COVID or Long-COVID Symptoms: Scoping Review. *J Med Internet Res, 25*, e45711. https://doi.org/10.2196/45711

Schmidt, S., Mühlan, H., & Power, M. (2005). The EUROHIS-QOL 8-item index: psychometric results of a cross-cultural field study. *European Journal of Public Health, 16*(4), 420-428. https://doi.org/10.1093/eurpub/cki155

Schröder, J., Bäuerle, A., Jahre, L. M., Skoda, E.-M., Stettner, M., Kleinschnitz, C., Teufel, M., & Dinse, H. (2023). Acceptance, drivers, and barriers to use eHealth interventions in patients with post-COVID-19 syndrome for management of post-COVID-19 symptoms: a cross-sectional study. *Therapeutic Advances in Neurological Disorders, 16*, 17562864231175730. https://doi.org/10.1177/17562864231175730

Soriano, J. B., Murthy, S., Marshall, J. C., Relan, P., Diaz, J. V., & Group, W. C. C. D. W. (2021). A clinical case definition of post-COVID-19 condition by a Delphi consensus. *The Lancet Infectious Diseases*.

Subramanian, A., Nirantharakumar, K., Hughes, S., Myles, P., Williams, T., Gokhale, K. M., Taverner, T., Chandan, J. S., Brown, K., Simms-Williams, N., Shah, A. D., Singh, M., Kidy, F., Okoth, K., Hotham, R., Bashir, N., Cockburn, N., Lee, S. I., Turner, G. M., Gkoutos, G. V., Aiyegbusi, O. L., McMullan, C., Denniston, A. K., Sapey, E., Lord, J. M., Wraith, D. C., Leggett, E., Iles, C., Marshall, T., Price, M. J., Marwaha, S., Davies, E. H., Jackson, L. J., Matthews, K. L., Camaradou, J., Calvert, M., & Haroon, S. (2022, 2022/08/01). Symptoms and risk factors for long COVID in non-hospitalized adults. *Nature Medicine, 28*(8), 1706-1714. https://doi.org/10.1038/s41591-022-01909-w

The Swiss Federal Council. Ordinance on Clinical Trials with the exception of Clinical Trials of Medical Devices. https://fedlex.data.admin.ch/filestore/fedlex.data.admin.ch/eli/cc/2013/643/20220526/en/pdf-a/fedlex-data-admin-ch-eli-cc-2013-643-20220526-en-pdf-a.pdf

Toussaint, A., Murray, A. M., Voigt, K., Herzog, A., Gierk, B., Kroenke, K., Rief, W., Henningsen, P., & Löwe, B. (2016). Development and Validation of the Somatic Symptom Disorder–B Criteria Scale (SSD-12). *Psychosomatic Medicine, 78*(1), 5-12. https://doi.org/10.1097/psy.0000000000000240

Üstün, T. B., Kostanjsek, N., Chatterji, S., & Rehm, J. (2010). *Measuring health and disability: Manual for WHO disability assessment schedule WHODAS 2.0*. World Health Organization.

WHO. (2021). *Coronavirus disease (COVID-19): Post COVID-19 condition* https://www.who.int/news-room/questions-and-answers/item/coronavirus-disease-(covid-19)-post-covid-19-condition

Williams, N. (2014). The GAD-7 questionnaire. *Occupational Medicine, 64*(3), 224-224. https://doi.org/10.1093/occmed/kqt161

Wise, J. (2022). Covid-19: WHO urges action as 17 million long covid cases are estimated in Europe. *BMJ, 378*, o2232. https://doi.org/10.1136/bmj.o2232

Wongvibulsin, S., Habeos, E. E., Huynh, P. P., Xun, H., Shan, R., Porosnicu Rodriguez, K. A., Wang, J., Gandapur, Y. K., Osuji, N., Shah, L. M., Spaulding, E. M., Hung, G., Knowles, K., Yang, W. E., Marvel, F. A., Levin, E., Maron, D. J., Gordon, N. F., & Martin, S. S. (2021, 2021/2/8). Digital Health Interventions for Cardiac Rehabilitation: Systematic Literature Review. *J Med Internet Res, 23*(2), e18773. https://doi.org/10.2196/18773

World Medical Association. (2013, Nov 27). *World Medical Association Declaration of Helsinki: ethical principles for medical research involving human subjects*. Retrieved Octoer 2022 from https://www.wma.net/policies-post/wma-declaration-of-helsinki-ethical-principles-for-medical-research-involving-human-subjects/

Ziauddeen, N., Gurdasani, D., O'Hara, M. E., Hastie, C., Roderick, P., Yao, G., & Alwan, N. A. (2022). Characteristics and impact of Long Covid: Findings from an online survey. *PLOS ONE, 17*(3), e0264331. https://doi.org/10.1371/journal.pone.0264331

Appendix 1: Schedule of assessments

	Outcomes/Parameters assessments	T0		Additional visits	T1	T2	T3
		1	2				
**0**	Oral and written information, written consent, screening inclusion-/exclusion criteria	x					
**1**	Routine clinical information/assessment	x		depending on clinical need			
**2**	Blood specimens (see Appendix 2)	x					x
**3**	Neurocognitive PC test battery: CNSVS	x	*				x
**4**	Psychometric Questionnaires						
a	Primary Outcome functional capacity: WHODAS 2.0 (12-item version)	x			x	x	x
b	VAS: quality of life, symptom intensity, functional impairment, work ability	x			x	x	x
c	Life quality: EuroHIS QOL-8	x			x	x	x
d	Symptoms: SSD-12, PHQ-15	x			x	x	x
e	Depression: PHQ-8	x			x	x	x
f	Anxiety: GAD-7	x			x	x	x
g	Resilience: RS-11	x			x	x	x
h	Insomnia: ISI	x			x	x	x
i	Fatigue: CFS	x			x	x	x
**5**	Measures of Physical Performance						
a	6 min walking test	x	*				x
b	Grip strength test (JAMAR® dynamometer)	x	*				x
c	st	x	*				x
**6**	Ecological momentary assessment (10 days after T0, T1, T2, and T3)		x		x	x	x
**7**	System Usability Scale				x		

Table 1. Schedule of assessments. Data from potential additional, merely clinical visits will be included in the study. The type and frequency of these visits are depending on each patients' clinical needs.

*: If the duration of the testing is too much of a strain for a patient to complete in one visit, a second visit on a separate day can be appointed.

CFS = Chalder Fatigue Scale, CNSVS = Central Nervous System Vital Signs, GAD = General Anxiety Disorder, ISI = Insomnia Severity Index, PHQ = Patient Health Questionnaire, RS = Resilience Scale, PSS = Perceived Stress Scale, QOL = Quality of Life, SSD = Somatic Symptom Disorder, VAS = visual analogue scale, WHODAS = World Health Organization Disability Assessment Schedule.

Appendix 2: Additional blood biospecimens to be shared with consortium partners (refer also to Joint Controllership Agreement)

- Biobank specimen (additional blood samples shipped to consortium partners, refer also to separate SOP protocols)
  - Biobank specimen #1 (B-Bio-0): **EDTA-plasma** 10 ml (DNA isolated from white blood cells, 15μl, concentration 50ng/μl)
    - Genome analyses at Helsinki University Hospital, Helsinki
    - HLA typing at Helsinki University Hospital, Finland
  - Biobank specimen #2 (S-Bio): **Serum**, 10 ml
    - COVID-19-antibody profile: send to HUS, Finland
    - Lipidome: send to Lipotype, Germany
    - Autoantibody epitopes: send to Protobios, Estonia
  - **Citrated plasma**: Double centrifuged, 10 ml, 4 aliquots (Patients with neurological symptoms or autoimmune features or thromboembolism)
    - Coagulation, send to HUS, Finland

Appendix 3: Screenshots from the smartphhone application and the platform

Smartphone application (patient’s view)

Platform (study personnel’s view)
